# Optical coherence tomography predictors of clinical outcomes after stent implantation: the ILUMIEN IV trial

**DOI:** 10.1093/eurheartj/ehae521

**Published:** 2024-08-28

**Authors:** Ulf Landmesser, Ziad A Ali, Akiko Maehara, Mitsuaki Matsumura, Richard A Shlofmitz, Giulio Guagliumi, Matthew J Price, Jonathan M Hill, Takashi Akasaka, Francesco Prati, Hiram G Bezerra, William Wijns, David Leistner, Paolo Canova, Fernando Alfonso, Franco Fabbiocchi, Giuseppe Calligaris, Rohit M Oemrawsingh, Stephan Achenbach, Carlo Trani, Balbir Singh, Robert J McGreevy, Robert W McNutt, Shih-Wa Ying, Jana Buccola, Gregg W Stone

**Affiliations:** Department of Cardiology, Angiology and Intensive Care Medicine, Deutsches Herzzentrum Charité, Charité—Universitätsmedizin Berlin, Berlin, Germany; Berlin Institute of Health, Berlin, Germany; German Centre for Cardiovascular Research, Berlin, Germany; St. Francis Hospital, Roslyn, NY, USA; Cardiovascular Research Foundation, New York, NY, USA; Cardiovascular Research Foundation, New York, NY, USA; Center for Interventional Cardiovascular Care, Columbia University, New York, NY, USA; Cardiovascular Research Foundation, New York, NY, USA; St. Francis Hospital, Roslyn, NY, USA; IRCCS Galeazzi Sant’Ambrogio Hospital, Milan, Italy; Division of Cardiovascular Diseases, Scripps Clinic, La Jolla, CA, USA; Royal Brompton Hospital, London, UK; Wakayama Medical University, Wakayama, Japan; Saint Camillus International University of Health Sciences, CLI Foundation, Rome, Italy; Tampa General Hospital, Tampa, FL; The Lambe Institute for Translational Medehance Spicine and Curam, University of Galway, Galway, Ireland; Department of Medicine, Cardiology, Goethe University Hospital, Frankfurt, Germany; German Center for Cardiovascular Research (DZHK) Partner Site RheinMain, Frankfurt, Germany; Ospedale Papa Giovanni XXIII, Bergamo, Italy; Cardiology Department, Hospital Universitario de La Princesa, CIBERCV, IIS-IP, Madrid, Spain; IRCCS Galeazzi Sant’Ambrogio Hospital, Milan, Italy; Department of Cardiovascular Sciences, University of Milan, Milano, Italy; Albert Schweitzer Ziekenhuis, Dordrecht, The Netherlands; Department of Cardiology, Friedrich-Alexander University Erlangen-Nürnberg, Erlangen, Germany; Fondazione Policlinico Universitario A. Gemelli, Rome, Italy; Max Super Specialty Hospital, Saket, New Delhi, India; Abbott Vascular, Santa Clara, CA, USA; Abbott Vascular, Santa Clara, CA, USA; Abbott Vascular, Santa Clara, CA, USA; Abbott Vascular, Santa Clara, CA, USA; The Zena and Michael A. Wiener Cardiovascular Institute, Icahn School of Medicine at Mount Sinai, 1 Gustave L. Levy Place, New York, NY 10029, USA

**Keywords:** Optical coherence tomography, Percutaneous coronary intervention, Stent, Prognosis

## Abstract

**Background and Aims:**

Observational registries have suggested that optical coherence tomography (OCT) imaging-derived parameters may predict adverse events after drug-eluting stent (DES) implantation. The present analysis sought to determine the OCT predictors of clinical outcomes from the large-scale ILUMIEN IV trial.

**Methods:**

ILUMIEN IV was a prospective, single-blind trial of 2487 patients with diabetes or high-risk lesions randomized to OCT-guided versus angiography-guided DES implantation. All patients underwent final OCT imaging (blinded in the angiography-guided arm). From more than 20 candidates, the independent OCT predictors of 2-year target lesion failure (TLF; the primary endpoint), cardiac death or target-vessel myocardial infarction (TV-MI), ischaemia-driven target lesion revascularization (ID-TLR), and stent thrombosis were analysed by multivariable Cox proportional hazard regression in single treated lesions.

**Results:**

A total of 2128 patients had a single treated lesion with core laboratory-analysed final OCT. The 2-year Kaplan–Meier rates of TLF, cardiac death or TV-MI, ID-TLR, and stent thrombosis were 6.3% (*n* = 130), 3.3% (*n* = 68), 4.3% (*n* = 87), and 0.9% (*n* = 18), respectively. The independent predictors of 2-year TLF were a smaller minimal stent area (per 1 mm^2^ increase: hazard ratio 0.76, 95% confidence interval 0.68–0.89, *P* < .0001) and proximal edge dissection (hazard ratio 1.77, 95% confidence interval 1.20–2.62, *P* = .004). The independent predictors of cardiac death or TV-MI were smaller minimal stent area and longer stent length; of ID-TLR were smaller intra-stent flow area and proximal edge dissection; and of stent thrombosis was smaller minimal stent expansion.

**Conclusions:**

In the ILUMIEN IV trial, the most important OCT-derived post-DES predictors of both safety and effectiveness outcomes were parameters related to stent area, expansion and flow, proximal edge dissection, and stent length.

## Structured Graphical Abstract

**Structured Graphical Abstract:**
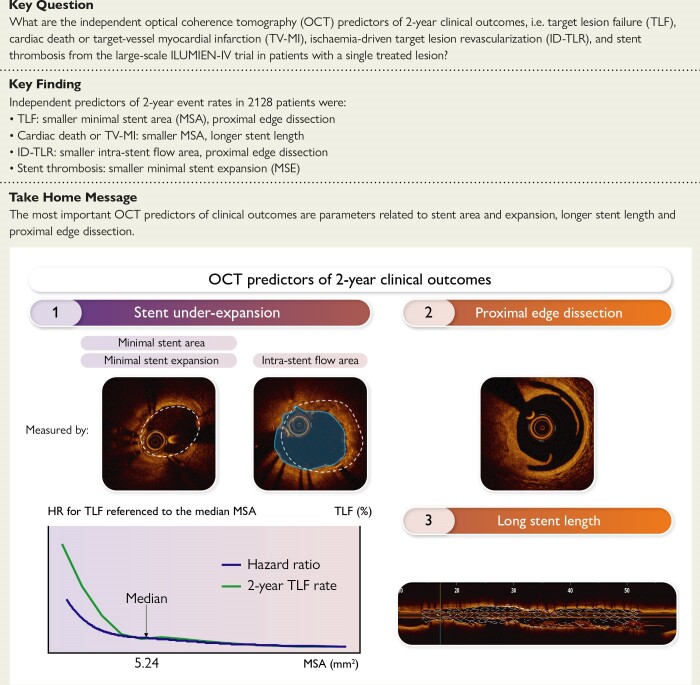
The independent optical coherence tomography predictors of clinical outcomes in 2128 patients with a single treated lesion in the ILUMIEN IV trial are shown here. Minimal stent area is defined as the smallest stent area within the contiguous stent segment. Minimal stent expansion is defined as the minimal stent area divided by the average of proximal and distal reference lumen areas × 100. Intra-stent flow (lumen) area is defined as stent area minus intra-stent plaque protrusion or thrombus area. HR, hazard ratio; ID-TLR, ischaemia-driven target lesion revascularization; MSA, minimal stent area; MSE, minimal stent expansion; OCT, optical coherence tomography; TLF, target lesion failure; TV-MI, target-vessel myocardial infarction

**See the editorial comment for this article ‘Optical coherence tomography for optimal stent implantation: what to check?’, by E. Romagnoli *et al.*, https://doi.org/10.1093/eurheartj/ehae626.**

## Introduction

The superior resolution of optical coherence tomography (OCT) compared with intravascular ultrasound (IVUS) leads to more accurate dimensional measurements and enhanced detection of suboptimal percutaneous coronary intervention (PCI) results including edge dissections, stent mal-apposition, and thrombus.^1^ However, not all of these findings are necessarily clinically relevant.^1–3^ Prior observational studies have identified variable OCT imaging predictors of adverse clinical outcomes after PCI^4–9^; however, the validity of these parameters is uncertain given the heterogeneity in study design and analyses and lack of prospective validation.

The OCT-Guided Coronary Stent Implantation Compared with Angiography: A Multicenter Randomized Trial in PCI (ILUMIEN IV: OPTIMAL PCI) was a prospective, randomized, single-blind trial in which 2487 patients with medication-treated diabetes or complex coronary artery lesions were randomly assigned to OCT-guided PCI or angiography-guided PCI.^10^ A novel aspect of ILUMIEN IV was the performance of a final OCT assessment in all patients, which was blinded to the operator in the angiography-guided group. Herein, we report a pre-specified analysis from the ILUMIEN IV trial examining the relationship between post-PCI OCT-derived parameters and subsequent clinical outcomes during 2-year follow-up after drug-eluting stent (DES) implantation.

## Methods

### Trial design and oversight

The prospective, single-blind, randomized ILUMIEN IV trial was conducted at 80 sites in 18 countries, including in North America, Europe, the Middle East, and Asia-Pacific region. The trial was approved by the institutional review board or ethics committee at each site, and all patients provided written informed consent. The sponsor (Abbott, Santa Clara, CA, USA) funded the trial and participated in site selection and data analysis. The principal investigators and study chair had unrestricted access to the data, prepared the manuscript, and attested to the accuracy of the analyses.

### Patients

Patients 18 years of age or older with evidence of myocardial ischaemia who were undergoing PCI and qualified as a high-risk patient or had one or more high-risk coronary artery lesions were enrolled. A high-risk patient was defined as having medication-treated diabetes mellitus. High-risk coronary lesions were defined as a lesion deemed responsible for a recent myocardial infarction; long or multiple lesions requiring >28 mm of stent; a bifurcation lesion with planned stenting of both the main and side branches; a severely calcified lesion; a chronic total occlusion; or diffuse or multifocal in-stent restenosis.

### Interventions

Eligible patients were randomly assigned in a 1:1 ratio to undergo OCT-guided or angiography-guided PCI with XIENCE cobalt chromium everolimus-eluting stents (Abbott). In the OCT-guided group, PCI was performed with a technique modified from the ILUMIEN III trial to allow optimization of stent sizing and length determination.^11^ A detailed description of the OCT-guidance and optimization algorithm has been published.^10,12^ Briefly, the distal reference mean external elastic lamina (EEL)-based diameter was measured and rounded down to the nearest available stent size in 0.25 mm increments to determine stent diameter. If the distal reference EEL could not be adequately visualized, the stent diameter was chosen using the mean lumen diameter at the distal reference rounded up to the next 0.25 mm stent size. Stent length was determined by the distance from distal to proximal reference site using the OCT-automated lumen detection feature. After stent deployment, optimization was performed with non-compliant balloons in the proximal and distal segments of the stent based on the respective EEL or lumen diameter measurements by rounding down (if EEL diameter was available) or up (if only lumen diameter was available) to the nearest non-compliant balloon size. Following optimization, OCT imaging was repeated and, if necessary, iterative high-pressure or larger non-compliant balloon inflations were performed, based on new reference segment measurements, to achieve acceptable stent expansion [a minimal stent area (MSA) of at least 90% in both the proximal and distal segments of the stent relative to the closest reference segment] if possible. The post-stent proximal and distal reference segments, defined as 5 mm from the edges of the stent, were also examined for inflow/outflow disease. If both the proximal and distal reference segments had a minimal lumen area (MLA) ≥ 4.5 mm^2^, no further treatment was deemed necessary. If there was untreated reference segment disease (defined as a focal MLA < 4.5 mm^2^ in either the proximal or distal reference segment), an additional DES was to be implanted unless anatomically prohibitive (e.g. biological vessel tapering, distal diffuse disease, and absence of landing zone). An additional DES was also recommended for treatment of a major edge dissection (defined as ≥60° of the circumference of the vessel at site of dissection and ≥3 mm in length).

A final OCT imaging run was performed at the end of the PCI procedure. For patients assigned to angiography guidance alone, these results were blinded to the operator.

### Optical coherence tomography analysis

Optical coherence tomography data were analysed at an independent imaging core laboratory [Cardiovascular Research Foundation (CRF), New York, NY, USA] without knowledge of randomized group. Optical coherence tomography images were considered not analysable if quantitative measurements were impaired from poor blood flush, significant non-uniform rotational distortion, or other reasons. All qualitative analyses were done using all available frames (0.2 mm frame intervals). All quantitative analyses were done every 1 mm. The key intra-stent OCT measurements were MSA, minimal stent expansion (MSE), intra-stent and total flow areas, major or minor tissue (plaque or thrombus) protrusion, major or minor stent mal-apposition, and stent deformation or fracture. The key stent edge-related OCT measurements were untreated reference segment disease that was further classified as focal or diffuse and lipidic or non-lipidic and major or minor dissection that was further categorized as intimal, medial (with or without haematoma), or adventitial. Detailed definitions of each of these parameters are provided in the Supplementary data.

### Clinical endpoints

Clinical follow-up was performed at 1, 12, and 24 months after the procedure; patients, healthcare providers, and outcomes assessors were blinded to treatment assignment group as previously described.^10,12^ Clinical events were adjudicated by an independent committee blinded to randomization assignment (CRF). The primary clinical endpoint for the current analysis was target lesion failure (TLF) defined as a composite of death from cardiac causes, target-vessel myocardial infarction (TV-MI) or ischaemia-driven target lesion revascularization (ID-TLR) at 2-year follow-up. Secondary endpoints were the composite of cardiac death or TV-MI, ID-TLR, and stent thrombosis (definite or probable). The definitions of these endpoints are described in detail in the Supplementary data.

### Statistical analysis

To be able to accurately relate lesion-specific post-PCI OCT parameters to events likely to have arisen from that lesion, the present analysis cohort was restricted to patients with a single treated coronary lesion who had a final OCT assessment that was analysable by the core laboratory. All analyses were performed on an intention-to-treat basis. Continuous variables are shown as mean ± standard deviation and were compared with a two-sample *t*-test. Categorical variables are shown as frequencies and were compared with a *χ*^2^ or Fisher's exact test. Time-to-first event estimates were generated with the Kaplan–Meier method and were compared using the log-rank test. Associations between OCT findings and clinical outcomes were evaluated using univariable and multivariable Cox proportional hazard regression and are presented as hazard ratios (HRs) with 95% confidence intervals (CIs). The functional form of each of the continuous variables was checked at the univariate level via martingale residuals, and no evidence of violation of the linearity assumption was detected. Variables that were eligible for inclusion in the multivariable models (i) had a univariable *P*-value of ≤.2; (ii) were present in ≥85% of the subjects in the analysis; (iii) had no subgroup with <30 patients (for categorical variables); and (iv) had the stronger statistical significance if highly correlated with another variable (|correlation coefficient| > 0.5). The associations between final MSA as a continuous variable and clinical outcomes are graphically presented with penalized spline curves with optimization of the degree of freedom using the Akaike information criteria based on the maximized log-likelihood of the model. All analyses were two sided, and *P*-values <.05 were considered significant. Missing data were not replaced. All statistical analyses were performed with SAS software, v9.4 (SAS Institute, Cary, NC, USA).

## Results

### Clinical and procedural characteristics

**Figure 1:**
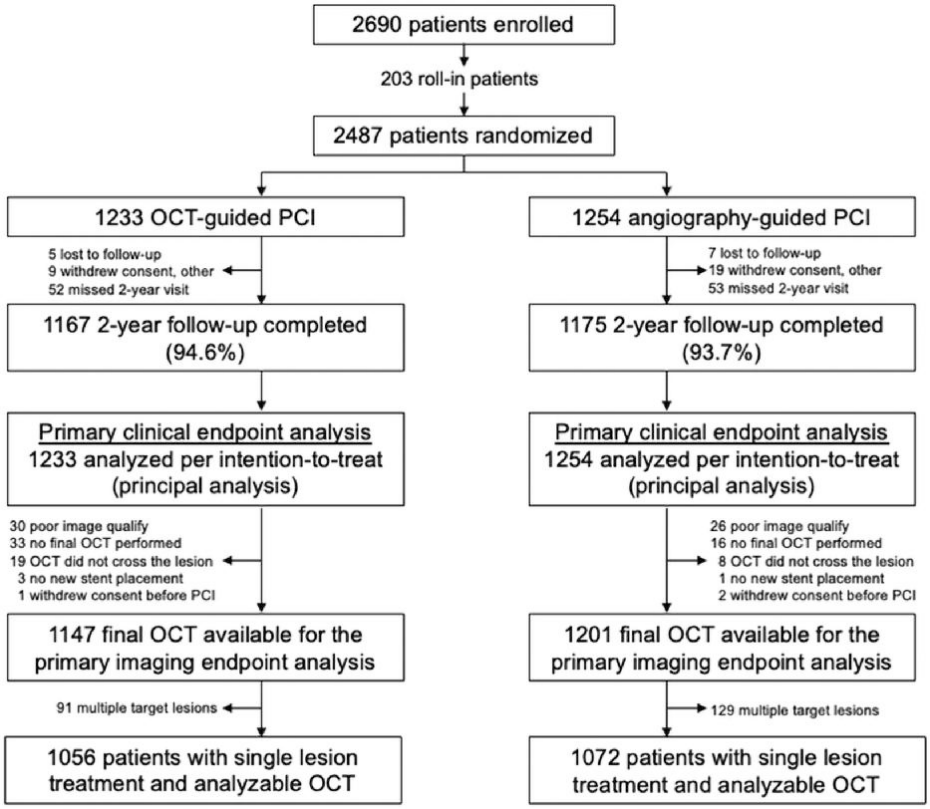
Consort diagram. The present analysis included randomized patients with single lesion treatment and core laboratory analysable final optical coherence tomography images. OCT, optical coherence tomography; PCI, percutaneous coronary intervention

A total of 2487 patients were randomized between 17 May 2018 and 29 December 2020, of whom 2128 (85.6%) had a single treated lesion with evaluable final OCT data that were included in the present analyses; 1056 patients were assigned to OCT-guided PCI and 1072 to angiography-guided PCI (*Figure 1*). Clinical and angiographic characteristics of patients included in the present analyses are shown in *Table 1* and Supplementary data online, *Table S1*, and procedural characteristics are shown in *Table 2*. Medication-treated diabetes was the qualifying high-risk patient characteristic in 39.2% of patients, and the most common qualifying high-risk lesion characteristics were long lesions (66.5%) and non-ST-segment elevation MI culprit lesion (23.6%). By angiographic core laboratory assessment, the mean reference vessel diameter was 2.93 ± 0.42 mm, the mean lesion length was 31.9 ± 15.9 mm, and 30.7% of lesions were severely calcified. All baseline characteristics were well matched between groups except for lesion length, which was longer in the OCT arm. Advanced lesion preparation, implanted stent length and diameter, number of post-dilatation balloons, and stent inflation pressures were higher in the OCT-guided group compared with the angiography-guided group. Procedure times, fluoroscopy duration, radiation dose, and contrast volume were also greater with OCT guidance. Medication use during follow-up is shown in Supplementary data online, *Table S2*.

**Table 1:** Demographic and clinical characteristics at baseline

	All (*N* = 2128)	OCT guidance (*n* = 1056)	Angiography guidance (*n* = 1072)	*P*-value
Age, years	65.3 ± 10.4	65.2 ± 10.5	65.4 ± 10.4	.68
Male sex	1637 (76.9)	827 (78.3)	810 (75.6)	.13
Race^a^				
White	1292/1643 (78.6)	648/819 (79.1)	644/824 (78.2)	.63
Black	67/1643 (4.1)	32/819 (3.9)	35/824 (4.2)	.73
Asian	268/1643 (16.3)	133/819 (16.2)	135/824 (16.4)	.94
Other	16/1643 (1.0)	6/819 (0.7)	10/824 (1.2)	.32
Hispanic or Latino ethnic group^a^	111/1597 (7.0)	53/797 (6.6)	58/800 (7.3)	.64
Hypertension	1527 (71.8)	743 (70.4)	784 (73.1)	.16
Dyslipidaemia	1411 (66.3)	684 (64.8)	727 (67.8)	.14
Diabetes mellitus	876 (41.2)	440 (41.7)	436 (40.7)	.64
Treated with any medication	834 (39.2)	416 (39.4)	418 (39.0)	.85
Treated with insulin	261 (12.3)	123 (11.6)	138 (12.9)	.39
Current smoker	427/2127 (20.1)	211 (20.0)	216/1071 (20.2)	.91
Body mass index, kg/m^2^	28.7 ± 5.3	28.6 ± 5.1	28.8 ± 5.4	.38
Previous myocardial infarction	472 (22.2)	216 (20.5)	256 (23.9)	.06
Previous PCI in the target vessel	269/2095 (12.8)	134/1035 (12.9)	135/1060 (12.7)	.89
Previous coronary artery bypass grafting	90 (4.2)	49 (4.6)	41 (3.8)	.35
Serum creatinine level, mg/dL^b^	0.95 ± 0.23	0.95 ± 0.23	0.96 ± 0.24	.62
Creatinine clearance, mL/min/1.73 m^2^b,c^^	79.2 ± 21.2	79.7 ± 21.1	78.8 ± 21.2	.37
<60 mL/min/1.73 m^2^	353/2067 (17.1)	161/1025 (15.7)	192/1042 (18.4)	.10
Dialysis	46 (2.2)	23 (2.2)	23 (2.1)	.96
Left ventricular ejection fraction, %	55.3 ± 8.6	55.2 ± 8.5	55.3 ± 8.6	.79
Clinical presentation				
Silent ischaemia	313 (14.7)	145 (13.7)	168 (15.7)	.21
Stable angina	610 (28.7)	296 (28.0)	314 (29.3)	.52
Unstable angina	579 (27.2)	291 (27.6)	288 (26.9)	.72
Non-STEMI	504 (23.7)	265 (25.1)	239 (22.3)	.13
Recent STEMI^d^	122 (5.7)	59 (5.6)	63 (5.9)	.77
Outpatient pharmacologic therapy				
Any antiplatelet therapy	1685 (79.2)	827 (78.3)	858 (80.0)	.33
Aspirin	1612 (75.8)	782 (74.1)	830 (77.4)	.07
P2Y_12_ antagonist	1065 (50.0)	534 (50.6)	531 (49.5)	.63
Dual antiplatelet therapy	992 (46.6)	489 (46.3)	503 (46.9)	.78
Beta-blocker	1148 (53.9)	585 (55.4)	563 (52.5)	.18
Calcium-channel blocker	467 (21.9)	232 (22.0)	235 (21.9)	.98
ACE inhibitor or ARB	1190 (55.9)	598 (56.6)	592 (55.2)	.51
Statin	1506 (70.8)	744 (70.5)	762 (71.1)	.75
Long-term oral anticoagulant	157 (7.4)	87 (8.2)	70 (6.5)	.13
Vitamin K antagonist	24 (1.1)	14 (1.3)	10 (0.9)	.39
Direct-acting oral anticoagulant	133 (6.3)	73 (6.9)	60 (5.6)	.21

Data are presented as means ± SD or *n* (%) or *n*/total *n* (%).

ACE, angiotensin-converting enzyme; ARB, angiotensin-receptor blocker; CABG, coronary bypass grafting; OCT, optical coherence tomography; PCI, percutaneous coronary intervention; STEMI, ST-elevation myocardial infarction.

^a^Data on race or ethnicity are missing from patients who declined to answer or were precluded from disclosing due to local regulations.

^b^Excluding patients on dialysis.

^c^Estimated using modification of diet in renal disease equation.

^d^Recent STEMI was defined as STEMI that had occurred within the previous 24 h.

**Table 2:** Patient and procedural characteristics

	All (*N* = 2128)	OCT guidance (*n* = 1056)	Angiography guidance (*n* = 1072)	*P*-value
Qualifying characteristics, per patient^a^				
Medication-treated diabetes	834/2125 (39.2)	416/1055 (39.4)	418/1070 (39.1)	.86
Culprit lesion non-STEMI	501/2125 (23.6)	262/1055 (24.8)	239/1070 (22.3)	.18
Culprit lesion STEMI^b^	119/2125 (5.6)	58/1055 (5.5)	61/1070 (5.7)	.84
Total stent length ≥28 mm^c^	1413/2125 (66.5)	718/1055 (68.1)	695/1070 (65.0)	.13
Two-stent bifurcation^d^	74/2125 (3.5)	37/1055 (3.5)	37/1070 (3.5)	.95
Severe calcification^e^	232/2125 (10.9)	109/1055 (10.3)	123/1070 (11.5)	.39
Chronic total occlusion^f^	138/2125 (6.5)	74/1055 (7.0)	64/1070 (6.0)	.33
Diffuse or multifocal in-stent restenosis	230/2125 (10.8)	114/1055 (10.8)	116/1070 (10.8)	.98
Procedural characteristics				
Advanced lesion preparation, per lesion^g^	221 (10.2)	135 (12.5)	86 (7.9)	.0004
Total stent length, per patient, mm	39.3 ± 20.6	41.6 ± 20.7	37.1 ± 20.2	<.0001
Maximum stent diameter, per lesion, mm	3.17 ± 0.43	3.22 ± 0.47	3.13 ± 0.39	<.0001
Post-dilatation performed, per lesion	1971 (91.1)	1044 (97.0)	927 (85.3)	<.0001
Post-dilatation balloons used, per lesion	1.5 ± 1.2	1.7 ± 1.1	1.4 ± 1.2	<.0001
Maximum device size, per lesion, mm	3.54 ± 0.52	3.69 ± 0.54	3.39 ± 0.46	<.0001
Maximum inflation pressure, per lesion, atm	19.2 ± 3.3	19.9 ± 3.1	18.4 ± 3.3	<.0001
Duration of procedure, per patient, min	56.0 ± 35.5	64.9 ± 35.8	47.3 ± 33.0	<.0001
Duration of fluoroscopy, per patient, min	18.1 ± 12.3	19.8 ± 13.0	16.6 ± 11.3	<.0001
Radiation dose, per patient, Gy	1.71 ± 1.55	1.94 ± 1.72	1.50 ± 1.34	<.0001
Contrast volume used per patient, mL	210.3 ± 83.3	227.8 ± 84.4	193.1 ± 78.6	<.0001

Data are presented as means ± SD or *n* (%) or *n*/total *n* (%).

OCT, optical coherence tomography; STEMI, ST-elevation myocardial infarction.

^a^Patients may have more than one qualifying characteristic.

^b^Data are shown for STEMIs that had occurred more than 24 h after symptom onset.

^c^The stent length reflects the continuous length or the total length of separated stents in any single-target vessel.

^d^Two-stent bifurcation was treatment with a stent at least 2.5 mm in diameter in both the main vessel and side-branch vessel.

^e^Severe calcification was defined as visible calcification on both sides of the vessel wall in the absence of cardiac motion.

^f^A chronic total occlusion could be a qualifying characteristic only after the occlusion had been successfully crossed with antegrade wire escalation and pre-dilatation.

^g^Advanced lesion preparation includes treatment with a cutting or scoring balloon, atherectomy, lithotripsy, or laser before implantation of the stent.

### Optical coherence tomography findings

The final post-PCI OCT findings in all patients and per randomized group are shown in *Table 3*. Optical coherence tomography guidance resulted in larger stent dimensions (including greater MSA, MSE, and intra-stent and total flow areas), less major but more minor tissue protrusion, less major mal-apposition, and fewer distal dissections (of all types) and fewer proximal and distal medial dissections (including fewer medial haematomas), compared with angiography guidance.

**Table 3:** Final optical coherence tomography results post-percutaneous coronary intervention, per lesion

	All (*N* = 2128)	OCT guidance (*n* = 1056)	Angiography guidance (*n* = 1072)	*P*-value
In-stent measures				
Minimal stent area, mm^2^	5.55 ± 1.94	5.74 ± 2.04	5.37 ± 1.81	<.0001
Minimal stent expansion, %	79.0 ± 17.0	80.7 ± 16.8	77.4 ± 17.0	<.0001
Minimal intra-stent flow area, mm^2^	5.42 ± 1.87	5.60 ± 1.96	5.25 ± 1.75	<.0001
Minimal total flow area, mm^2^	5.56 ± 1.90	5.73 ± 1.99	5.40 ± 1.79	<.0001
Stent length, mm	35.8 ± 17.4	37.6 ± 17.1	34.0 ± 17.5	<.0001
Plaque or thrombus protrusion, any	1094 (51.4)	593 (56.2)	501 (46.7)	<.0001
Major	147 (6.9)	55 (5.2)	92 (8.6)	.002
Minor	947 (44.5)	538 (50.9)	409 (38.2)	<.0001
Mal-apposition, any	1362 (64.0)	591 (56.0)	771 (71.9)	<.0001
Major	549/2127 (25.8)	175/1055 (16.6)	374/1072 (34.9)	<.0001
Minor	812/2127 (38.2)	415/1055 (39.3)	397/1072 (37.0)	.27
Stent deformation or fracture	21 (1.0)	12 (1.1)	9 (0.8)	.49
Peri-stent (edge) measures				
Proximal or distal reference disease, any	513/2121 (24.2)	242/1052 (23.0)	271/1069 (25.4)	.21
Focal	326/2121 (15.4)	150/1052 (14.3)	176/1069 (16.5)	.16
Diffuse	210/2121 (9.9)	103/1052 (9.8)	107/1069 (10.0)	.87
Proximal reference disease, any	230/1904 (12.1)	108/933 (11.6)	122/971 (12.6)	.51
Focal	170/1904 (8.9)	79/933 (8.5)	91/971 (9.4)	.49
Diffuse	60/1904 (3.2)	29/933 (3.1)	31/971 (3.2)	.92
Distal reference disease, any	343/2089 (16.4)	155/1030 (15.0)	188/1059 (17.8)	.10
Focal	177/2089 (8.5)	76/1030 (7.4)	101/1059 (9.5)	.08
Diffuse	166/2089 (7.9)	79/1030 (7.7)	87/1059 (8.2)	.64
Lipidic plaque	355/2122 (16.7)	170/1053 (16.1)	185/1069 (17.3)	.47
Proximal reference	238/1921 (12.4)	115/943 (12.2)	123/978 (12.6)	.80
Distal reference	144/2094 (6.9)	67/1035 (6.5)	77/1059 (7.3)	.47
Proximal or distal reference dissection, any	695/2125 (32.7)	329/1054 (31.2)	366/1071 (34.2)	.15
Major	66/2125 (3.1)	25/1054 (2.4)	41/1071 (3.8)	.053
Minor	629/2125 (29.6)	304/1054 (28.8)	325/1071 (30.3)	.45
Intimal	264/2125 (12.4)	132/1054 (12.5)	132/1071 (12.3)	.89
Medial	460/2125 (21.6)	208/1054 (19.7)	252/1071 (23.5)	.03
Haematoma	71/2125 (3.3)	27/1054 (2.6)	44/1071 (4.1)	.047
No haematoma	398/2125 (18.7)	185/1054 (17.6)	213/1071 (19.9)	.17
Adventitial	3/2125 (0.1)	1/1054 (0.1)	2/1071 (0.2)	1.00
Proximal reference dissection, any	418/1997 (20.9)	215/984 (21.8)	203/1013 (20.0)	.32
Major	34/1997 (1.7)	13/984 (1.3)	21/1013 (2.1)	.19
Minor	384/1997 (19.2)	202/984 (20.5)	182/1013 (18.0)	.15
Intimal	188/1997 (9.4)	102/984 (10.4)	86/1013 (8.5)	.15
Medial	229/1997 (11.5)	113/984 (11.5)	116/1013 (11.5)	.98
Haematoma	25/1997 (1.3)	12/984 (1.2)	13/1013 (1.3)	.90
No haematoma	204/1997 (10.2)	101/984 (10.3)	103/1013 (10.2)	.94
Adventitial	1/1997 (0.1)	0/984 (0.0)	1/1013 (0.1)	1.00
Distal reference dissection, any	371/2109 (17.6)	160/1043 (15.3)	211/1066 (19.8)	.007
Major	32/2109 (1.5)	12/1043 (1.2)	20/1066 (1.9)	.17
Minor	339/2109 (16.1)	148/1043 (14.2)	191/1066 (17.9)	.02
Intimal	89/2109 (4.2)	38/1043 (3.6)	51/1066 (4.8)	.19
Medial	280/2109 (13.3)	121/1043 (11.6)	159/1066 (14.9)	.02
Haematoma	46/2109 (2.2)	15/1043 (1.4)	31/1066 (2.9)	.02
No haematoma	234/2109 (11.1)	106/1043 (10.2)	128/1066 (12.0)	.18
Adventitial	2/2109 (0.1)	1/1043 (0.1)	1/1066 (0.1)	1.00

Data are presented as mean ± SD or *n* (%) or *n*/total *n* (%) if the denominator is different than the denominator at the top of the column (%).

OCT, optical coherence tomography; PCI, percutaneous coronary intervention.

### Clinical endpoints

The 2-year Kaplan–Meier rates of TLF, cardiac death or TV-MI, ID-TLR, and stent thrombosis were 6.3% (*n* = 130), 3.3% (*n* = 68), 4.3% (*n* = 87), and 0.9% (*n* = 18), respectively. Similar to the principal results from the entire trial,^10^ event rates in this study cohort were not significantly different between the randomized groups, except for stent thrombosis that occurred less frequently after OCT guidance compared with angiography guidance (*Table 4*).

**Table 4:** Clinical outcomes at 2 years

	All (*N* = 2128)	OCT guidance (*n* = 1056)	Angiography guidance (*n* = 1072)	Hazard ratio (95% CI)	*P*-value
Target lesion failure	130 (6.3)	63 (6.2)	67 (6.4)	0.95 (0.67–1.34)	.76
Cardiac death or TV-MI	68 (3.3)	30 (2.9)	38 (3.6)	0.80 (0.49–1.29)	.35
Cardiac death	20 (1.0)	7 (0.7)	13 (1.3)	0.54 (0.22–1.36)	.18
Target-vessel MI	54 (2.6)	24 (2.3)	30 (2.9)	0.81 (0.47–1.38)	.43
Ischaemia-driven TLR	87 (4.3)	45 (4.5)	42 (4.1)	1.08 (0.71–1.64)	.73
Stent thrombosis, definite or probable	18 (0.9)	4 (0.4)	14 (1.3)	0.29 (0.10–0.88)	.02

Outcomes are presented as number of events (Kaplan–Meier estimated event rates).

CI, confidence interval; TV-MI, target-vessel myocardial infarction; OCT, optical coherence tomography; TLR, target lesion revascularization.

### Optical coherence tomography predictors of clinical outcomes

**Figure 2:**
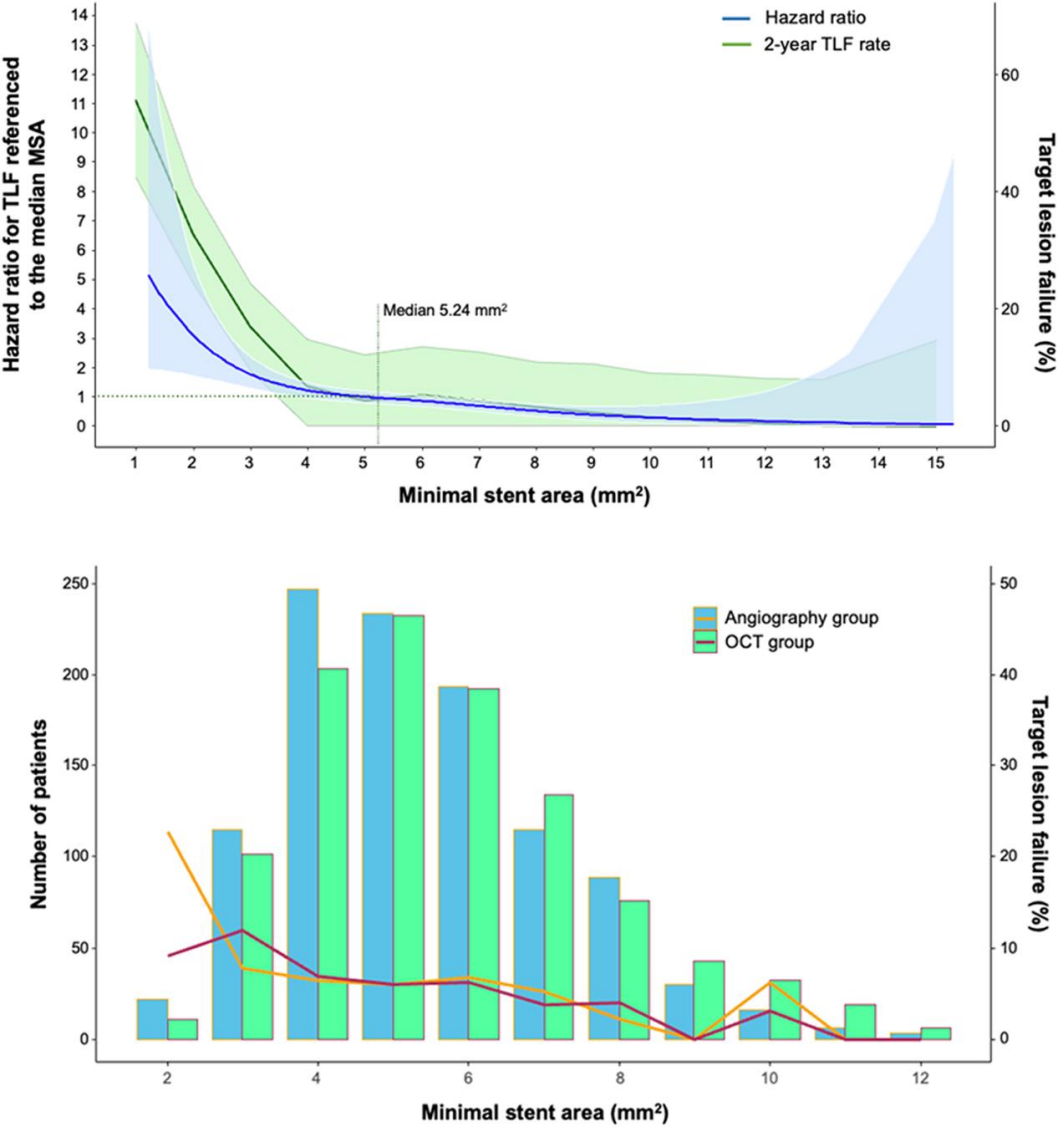
Distribution of optical coherence tomography-derived minimal stent area and its relationship with target lesion failure during 2-year follow-up. Top: Penalized spline analysis showing the 2-year target lesion failure rate (green line) and 95% confidence interval (green shading) in the pooled optical coherence tomography and angiography guidance groups for each post-percutaneous coronary intervention minimal stent area value as a continuous measure. The blue line and shading represents the penalized spline relationship of the hazard ratio and 95% confidence interval for target lesion failure for each minimal stent area value referenced to a hazard ratio of 1.0 for the median minimal stent area of 5.24 mm^2^. Note that the hazard ratio is calculated from the actual continuous minimal stent area value, which ranged from 1.22 to 15.30 mm^2^. In contrast, the target lesion failure rate is based on a rounding function such that minimal stent area of 1.22 is rounded to 1 and 15.30 is rounded to 15. As such, the curves appear slightly offset. Bottom: Histogram bars showing the numbers of patients in each randomized group (left *y*-axis) with minimal stent area in 1 mm increments centred around each whole number (e.g. the bars to the left and right of the 2 mm^2^ minimal stent area tick mark includes minimal stent area values ranging from 1.5 to 2.5 mm^2^). The right *y*-axis shows the 2-year rate of target lesion failure (line graphs) for each associated minimal stent area grouping in patients randomized to optical coherence tomography guidance vs. angiography guidance. MSA, minimal stent area; OCT, optical coherence tomography; TLF, target lesion failure

As shown in *Table 5*, the univariable (unadjusted) OCT correlates of 2-year TLF were smaller stent dimensions (including MSA, MSE, intra-stent flow area, and total flow area) and proximal reference segment dissection, especially medial and minor dissections. By multivariable analysis, the independent predictors of 2-year TLF were smaller MSA (per 1 mm^2^ increase: HR 0.76, 95% CI 0.68–0.86, *P* < .0001) and proximal edge dissection (HR 1.77, 95% CI 1.20–2.62, *P* = .004) (*Table 6*). The distribution and relationship between MSA and the 2-year rate of TLF in the randomized groups are shown in *Figure 2*. A non-linear relationship was present wherein the rate of TLF increased exponentially below an MSA of 4.0 mm^2^.

**Table 5:** Optical coherence tomography findings associated with target lesion failure (unadjusted)

	Event rate	Hazard ratio (95% CI)	*P*-value
Minimal stent area, mm^2^			
<5.24 (median)	81/1063 (7.6)	Reference	
≥5.24	49/1065 (4.6)	0.59 (0.42–0.85)	.004
Minimal stent expansion, %			
<79.1 (median)	78/1063 (7.3)	Reference	
≥79.1	52/1065 (4.9)	0.65 (0.46–0.93)	.02
Intra-stent flow area, mm^2^			
<5.14 (median)	82/1061 (7.7)	Reference	
≥5.14	48/1067 (4.5)	0.57 (0.40–0.81)	.002
Total flow area, mm^2^			
<5.23 (median)	82/1058 (7.8)	Reference	
≥5.23	48/1070 (4.5)	0.57 (0.40–0.81)	.002
Stent length, mm			
<33.4 (median)	58/1056 (5.5)	Reference	
≥33.4	72/1072 (6.7)	1.24 (0.88–1.76)	.22
Plaque or thrombus protrusion			
None	63/1034 (6.1)	Reference	
Any	67/1094 (6.1)	1.00 (0.71–1.41)	1.00
Major	11/147 (7.5)	1.22 (0.64–2.31)	.55
Minor	56/947 (5.9)	0.97 (0.67–1.39)	.85
Mal-apposition			
None	48/766 (6.3)	Reference	
Any	82/1362 (6.0)	0.96 (0.67–1.37)	.83
Major	38/549 (6.9)	1.11 (0.73–1.70)	.62
Minor	44/812 (5.4)	0.86 (0.57–1.30)	.48
Stent deformation or fracture			
None	128/2107 (6.1)	Reference	
Any	2/21 (9.5)	1.50 (0.37–6.07)	.57
Proximal or distal reference disease			
None	91/1608 (5.7)	Reference	
Any	39/513 (7.6)	1.36 (0.93–1.98)	.11
Focal	24/326 (7.4)	1.31 (0.84–2.06)	.23
Diffuse	18/210 (8.6)	1.54 (0.93–2.55)	.10
Proximal reference disease			
None	87/1674 (5.2)	Reference	
Any	19/230 (8.3)	1.63 (0.99–2.68)	.053
Focal	14/170 (8.2)	1.62 (0.92–2.85)	.09
Diffuse	5/60 (8.3)	1.66 (0.67–4.09)	.27
Distal reference disease			
None	100/1746 (5.7)	Reference	
Any	26/343 (7.6)	1.34 (0.87–2.06)	.19
Focal	11/177 (6.2)	1.09 (0.58–2.03)	.79
Diffuse	15/166 (9.0)	1.60 (0.93–2.76)	.09
Proximal or distal reference lipidic plaque			
None	114/1767 (6.5)	Reference	
Any	16/355 (4.5)	0.69 (0.41–1.17)	.17
Proximal reference lipidic plaque			
None	100/1683 (5.9)	Reference	
Any	9/238 (3.8)	0.63 (0.32–1.25)	.19
Distal reference lipidic plaque			
None	119/1950 (6.1)	Reference	
Any	7/144 (4.9)	0.79 (0.37–1.70)	.55
Proximal or distal reference dissection			
None	81/1430 (5.7)	Reference	
Any	49/695 (7.1)	1.26 (0.88–1.79)	.21
Major	4/66 (6.1)	1.06 (0.39–2.89)	.91
Minor	45/629 (7.2)	1.28 (0.89–1.84)	.19
Intimal	17/264 (6.4)	1.15 (0.68–1.94)	.60
Medial	36/460 (7.8)	1.40 (0.94–2.07)	.10
Haematoma	7/71 (9.9)	1.75 (0.81–3.79)	.16
No haematoma	30/398 (7.5)	1.35 (0.89–2.05)	.16
Adventitial	0/3 (0.0)	Not applicable	.98
Proximal reference dissection, any			
None	79/1579 (5.0)	Reference	
Any	37/418 (8.9)	1.80 (1.22–2.66)	.003
Major	3/34 (8.8)	1.81 (0.57–5.72)	.32
Minor	34/384 (8.9)	1.80 (1.21–2.69)	.004
Intimal	16/188 (8.5)	1.74 (1.02–2.97)	.04
Medial	21/229 (9.2)	1.86 (1.15–3.01)	.01
Haematoma	3/25 (12.0)	2.43 (0.77–7.68)	.13
No haematoma	18/204 (8.8)	1.79 (1.08–2.99)	.03
Adventitial	0/1 (0.0)	Not applicable	.99
Distal reference dissection			
None	107/1738 (6.2)	Reference	
Any	19/371 (5.1)	0.84 (0.51–1.36)	.47
Major	1/32 (3.1)	0.49 (0.07–3.48)	.47
Minor	18/339 (5.3)	0.87 (0.53–1.43)	.58
Intimal	1/89 (1.1)	0.18 (0.03–1.29)	.09
Medial	18/280 (6.4)	1.05 (0.64–1.74)	.84
Haematoma	4/46 (8.7)	1.42 (0.52–3.85)	.49
No haematoma	14/234 (6.0)	0.98 (0.56–1.71)	.95
Adventitial	0/2 (0.0)	Not applicable	.98

All subcategories are compared with the reference. Outcomes are presented as number of events during 2-year follow-up/number of the patients at risk on Day 0 (Kaplan–Meier estimated event rates).

CI, confidence interval; OCT, optical coherence tomography.

**Table 6:** Optical coherence tomography findings independently associated with clinical endpoints (adjusted analysis)

Outcome	*N*	OCT variable	Hazard ratio (95% CI)	*P*-value
Target lesion failure	1997	Minimal stent area, per 1 mm^2^	0.76 (0.68–0.86)	<.0001
		Proximal edge dissection, any	1.77 (1.20–2.62)	.004
Cardiac death or TV-MI	2128	Minimal stent area, per 1 mm^2^	0.82 (0.70–0.95)	.009
		Stent length, per 5 mm	1.08 (1.02–1.15)	.009
Ischaemia-driven TLR	1997	Intra-stent flow area, per 1 mm^2^	0.72 (0.62–0.84)	<.0001
		Proximal edge dissection, any	1.88 (1.16–3.03)	.01
		Plaque or thrombus protrusion, major	1.95 (0.97–3.92)	.06
Stent thrombosis	2128	Minimal stent expansion, per 10%	0.71 (0.55–0.93)	.01

*N*, the number of patients included in each analysis model.

CI, confidence interval; OCT, optical coherence tomography; ref, reference; TLR, target lesion revascularization; TV-MI, target vessel myocardial infarction.

The unadjusted correlates of cardiac death or TV-MI, ID-TLR, and stent thrombosis are shown in Supplementary data online, *Tables S3*–*S5*. As shown in *Table 6*, the independent predictors of cardiac death or TV-MI were smaller MSA (per 1 mm^2^ increase: HR 0.82, 95% CI 0.70–0.95, *P* = .009) and longer stent length (per 5 mm increase: HR 1.08, 95% CI 1.02–1.15, *P* = .009); of ID-TLR were smaller intra-stent flow area (per 1 mm^2^ increase: HR 0.72, 95% CI 0.62–0.84, *P* < .0001) and proximal edge dissection (HR 1.88, 95% CI 1.16–3.03, *P* = .01), with major tissue protrusion being of borderline significance (HR 1.95, 95% CI 0.97–3.92, *P* = .06); and of stent thrombosis was smaller MSE (per 10% increase: HR 0.71, 95% CI 0.55–0.93, *P* = .01). The number and percentage of missing values for these analysed variables are shown in Supplementary data online, *Table S6*.

## Discussion

In the present pre-specified analysis from ILUMIEN IV, the largest, prospective, randomized trial to date of intravascular imaging guidance vs. angiography guidance for PCI, we examined the immediate post-PCI OCT imaging predictors of clinical outcomes during a 2-year follow-up period in patients with a single treated lesion. The main findings of the present analysis are as follows. First, OCT guidance led to greater mean MSA, MSE, and intra-stent and total flow areas compared with angiography guidance alone. Optical coherence tomography guidance also reduced the frequency of post-PCI major tissue protrusion, major mal-apposition, proximal and distal medial dissections (including fewer medial haematomas), and distal dissections of all types. Second, only smaller MSA and the presence of a proximal edge dissection independently predicted the occurrence of TLF during follow-up. Third, the only independent OCT predictors of the composite of cardiac death or TV-MI were smaller MSA and stent length; of ID-TLR were intra-stent flow area and proximal edge dissection; and of stent thrombosis was smaller MSE (*Structured Graphical Abstract*).

As summarized in Supplementary data online, *Table S7*, prior studies have examined the association between intravascular imaging findings and clinical outcomes. Most of these studies were retrospective, many were single centre, not all used an independent core laboratory, and all were substantially smaller than the present prospective, multi-centre trial. Nonetheless, a smaller MSA has consistently been identified as the strongest predictor of future adverse events after DES implantation.^7,8,13–16^ Based on these findings, the current European consensus document on intravascular imaging guidance of PCI endorses a MSE ≥ 80% and/or MSA > 4.5 mm^2^ by OCT as acceptable.^2^ Notably, the median MSE was 79.1% in the present study, and patients with a treated lesion above this median had a lower TLF rate and a lower rate of cardiac death or TV-MI, supporting the European Association of Percutaneous Cardiovascular Interventions recommendations. The present findings strongly reinforce the concept that achieving larger absolute stent areas and greater relative stent expansion should remain the primary target for OCT-guided PCI optimization. We found a smaller MSA to be an independent predictor not only of TLF but also of the composite outcome of cardiac death or TV-MI. Correspondingly, we found that the intra-stent flow area, a variable co-linear with MSA, independently predicted ID-TLR, and MSE, another variable co-linear with MSA, independently predicted stent thrombosis. The present analysis does not afford a ready explanation as to why one or the other variable (MSA vs. MSE vs. intra-stent flow area) that was all entered into each multivariable analysis as covariates was the strongest independent predictor of the different safety and effectiveness outcomes after DES. Nonetheless, they all point to the consistent conclusion that optimizing stent dimensions and expansion with OCT guidance is associated with subsequent freedom from stent-related adverse events.

In this regard, in the ILUMIEN IV trial, DES implanted with OCT guidance had a larger mean MSA compared with angiography guidance alone, but paradoxically, this did not result in a significant reduction in the 2-year rate of the primary study outcomes of target vessel failure (HR 0.90).^10^ Specifically, while there were numerically fewer cardiac deaths and TV-MIs and significantly fewer stent thromboses with OCT guidance, the rates of ID-TLR in the OCT-guided and angiography-guided groups were almost identical.^10^ One potential explanation for the discordance between the different group mean MSAs and non-significant difference in target vessel failure (or TLF in the present analysis) relates to the absolute MSAs achieved. Spline analysis from the present study demonstrated a steep and exponential risk of TLF with decreasing OCT-MSA below 4 mm^2^. However, the post-PCI mean MSAs achieved in both groups in ILUMIEN IV (OCT 5.72 ± 2.04 mm^2^; angiography 5.36 ± 1.87 mm^2^)^11^ were greater than in previous studies that reported an optimal MSA cut-off for DES of 4.5–5.3 mm^2^ (see Supplementary data online, *Table S7*). In part, this may be explained by the fact that the mean maximum device size was larger in the present trial (3.54 ± 0.52 mm) than in most prior studies, and inflation pressures were notably higher, even in the control arm. Thus, while OCT did result in fewer patients with post-PCI MSA < 4 mm^2^ than angiography guidance (as seen in *Figure 2*), the absolute proportion of patients favouring OCT guidance in this critical range may not have been sufficient to drive a significant difference in outcomes (other than for stent thrombosis). In this regard, a small MSE was a stronger predictor of stent thrombosis than a small MSA. Since an adequate MSA can still be achieved with stent under-expansion, this finding highlights the potential aetiologic importance of abnormal rheology on stent thrombosis.

The high resolution of OCT identifies peri-stent dissections in up to 40% of cases,^11^ ∼80% undetectable by angiography, and many of which may heal without consequence.^17–19^ As such, identifying which dissections lead to adverse clinical outcomes is important. Some studies have identified major stent edge dissections detected by OCT as a predictor of poor outcomes (see Supplementary data online, *Table S7*).^6,8,9,19^ In the CLI-OPCI registry, a dissection with width > 200 μm at the distal edge (but not at the proximal edge) was associated with a 2.5-fold hazard of major adverse cardiovascular events (MACE).^6,8^ In a detailed analysis of OCT-identified edge dissections, van Zandvoort *et al*.^19^ reported that cavity depth at the distal edge, reference lumen area at the proximal edge, and overall dissection length were predictors of MACE. In contrast, the present larger study identified proximal edge but not distal edge dissections as an independent predictor of TLF and ID-TLR. Importantly, this finding was independent of untreated reference segment disease (at either edge), which was not retained in the multivariable model. In addition, we were unable to demonstrate that major dissections were of greater risk than minor dissections, although by univariable analysis medial dissections, especially when a haematoma was visualized by OCT, may portend greater risk. However, even ILUMIEN IV did not contain enough dissections of each type to state with certainty, which are of greatest clinical relevance, a topic warranting future studies with even larger OCT databases. Until that time, we believe that treating major proximal (and distal) dissections as identified by OCT is prudent. Finally, also of note is the finding that acute stent mal-apposition was not an independent predictor of adverse outcomes, a finding consistent with nearly all prior studies.^20^ As such, while stent mal-apposition is often a dramatic finding on OCT (often more so than with IVUS), most cases of acute stent mal-apposition do not require treatment unless associated with a small MSA or stent under-expansion.

### Limitations

To isolate the effect of post-PCI OCT parameters on outcomes, we restricted our analyses to cases with a single treated lesion, potentially excluding higher-risk patients. Moreover, the present analysis focused on lesion-specific OCT predictors of clinical outcomes and not on vessel or patient predictors. Also, the OCT treatment algorithm mandated treatment of major mal-apposition, tissue protrusion, and major dissection, resulting in ∼25%–50% fewer such findings in the OCT arm. Further detailed analysis of the impact of these untreated conditions in the two arms (which may be of different severity) is warranted and is underway. However, as shown herein, the relationship between post-PCI MSA and TLF was consistent after OCT guidance and angiography guidance. Because IVUS often overestimates quantitative measures compared with OCT,^21^ the critical values for OCT-MSA described in the present report cannot be translated to IVUS guidance. Notably, operators detected suboptimal OCT findings, such as stent expansion < 90%, untreated reference segment disease, dissection, mal-apposition, and tissue protrusion at a lower rate compared with the core lab (see Supplementary data online, *Table S8*), highlighting the potential importance of incorporating artificial intelligence algorithms into OCT platforms. Another potential limitation of OCT guidance is the lack of data on coronary physiology. However, OCT-based virtual fractional flow reserve systems have been developed, and the combination of OCT-derived imaging and functional data may provide synergistic benefits. Finally, the COVID-19 pandemic may have reduced elective revascularization procedures in patients with recurrent angina or ischaemia, contributing to the low and similar rates of ID-TLR in the randomized groups.^10^ This issue did not have a major effect on the present study in which the relationship between final OCT findings and clinical outcomes in both the OCT-guided and angiography-guided study arms was analysed together. We cannot, however, exclude the possibility that additional predictors of clinical outcomes might have been detected had the study sample size been larger.

In conclusion, the present study, the largest analysis to date of intravascular imaging-derived predictors of clinical outcomes after PCI demonstrates that both a smaller absolute MSA and lower relative stent expansion are strongly associated with adverse safety and effectiveness outcomes after coronary DES implantation. Proximal edge dissections as detected by OCT were also an independent predictor of TLF and ID-TLR. Additional studies are warranted to determine whether optimizing these parameters with OCT guidance to an even greater extent than was achieved in ILUMIEN IV may further improve DES outcomes.

## Supplementary data

Supplementary data are available at *European Heart Journal* online.

## Supplementary Material

ehae521_Supplementary_Data

## Declarations

### Data Availability

The data, methods used in the analysis, and materials used to conduct the research will not be made publicly available. The authors will provide data for reproducing the results for the purposes of audit or governance under a confidentiality agreement. Any relevant inquiry should be emailed to the study chairman G.W.S. (Email: gregg.stone@mountsinai.org).

### Pre-registered Clinical Trial Number

The pre-registered clinical trial number is ClinicalTrials.gov NCT03507777.
