# Supplementary material for: Optical coherence tomography predictors of clinical outcomes after stent implantation: the ILUMIEN IV trial
Source: Eur Heart J. 2024 Aug 28;45(43):4630–43. doi: 10.1093/eurheartj/ehae521 (PMC11560276; doi:10.1093/eurheartj/ehae521)
Supplement: ehae521_Supplementary_Data [file ehae521_supplementary_data.docx]

**Supplementary Appendix**

**Table of Contents**

|  | | **Page** |
| --- | --- | --- |
| Definitions of optical coherence tomography-derived parameters | | **2** |
| Definitions of clinical endpoints | | **4** |
| Table S1 | Angiographic characteristics and outcomes (core laboratory-assessed) | **7** |
| Table S2 | Medication use during study follow-up | **8** |
| Table S3 | OCT findings associated with cardiac death or target vessel myocardial infarction (unadjusted) | **10** |
| Table S4 | OCT findings associated with ischemic-driven target lesion revascularization (unadjusted) | **12** |
| Table S5 | OCT findings associated with definite or probable stent thrombosis (unadjusted) | **14** |
| Table S6 | Numbers and percentages of missing values in analyzed variables | **16** |
| Table S7 | Summary of prior studies examining the association between intravascular imaging parameters and clinical outcomes | **17** |
| Table S8 | Comparison of operator-detected vs. core laboratory-detected OCT findings | **22** |
| Figure S1 | Distribution of OCT-derived minimal stent expansion and its relationship with target lesion failure (TLF) during 2-year follow-up | **23** |
| References | | **25** |

**Definitions of Optical Coherence Tomography-Derived Parameters**

1. Minimal stent area (MSA) (mm^2^) (continuous variable): The smallest stent area within the contiguous stent segment
2. Minimal stent expansion (%) (continuous variable): The MSA divided by the average of proximal and distal reference lumen areas × 100.

Reference site was defined as the largest lumen area within 5mm from the stent edge before significant side branch (lumen diameter≥1.5mm). In case without any reference (i.e. The stent ended at the ostium), the last frame of the stent area before bifurcation was used as the reference lumen area.

1. Intra-stent flow area (intra-stent lumen area) (continuous variable): Defined as stent area minus intra-stent plaque protrusion or thrombus.
2. Total flow area (total lumen area) (continuous variable): Defined as intra-stent lumen area plus any area of malapposition between the stent and the vessel wall (lumen border/plaque surface).
3. Intra-stent tissue protrusion (plaque or thrombus) (categorical variable): A mass attached to the luminal surface or floating within the lumen, and at least 0.2 mm in diameter within the luminal edge of a stent strut, and will be further classified as Major and Minor:
   - - Major: Protrusion area/Stent area at site of tissue protrusion ≥10% and the minimal intrastent flow area (MSA – protrusion area) is unacceptable (<90% of respective proximal or distal reference area
     - Minor: Protrusion area/Stent area at site of tissue protrusion is <10%, or is ≥10% but the minimal intraluminal flow area (MSA – protrusion area) is acceptable (≥90% of respective proximal or distal reference area

Note: It was recommended that if protrusion was detected by operator assessment in the OCT-guided arm during the procedure and met the criteria for major protrusion, then thrombus aspiration, further high-pressure balloon inflation and/or an additional stent be considered.

1. Stent malapposition (categorical variable): Defined as stent struts clearly separated from the vessel wall (lumen border/plaque surface) without any tissue behind the struts with a distance from the adjacent intima of ≥0.2 mm and not associated with any side branch, and will be further classified as Major and Minor:
   - - Major: if associated with unacceptable stent expansion (MSA <90% of respective proximal or distal reference lumen area)
     - Minor: if associated with acceptable stent expansion (MSA ≥90% of respective proximal or distal reference lumen area)

Note: If malapposition was detected by operator assessment during the procedure in the OCT-guided arm and met the criteria for major malapposition (i.e. malapposition associated with unacceptable stent expansion), the protocol directed that further stent expansion must be performed. The degree of stent under-expansion (acceptable or unacceptable) should guide the intervention rather than amount of malapposition.

1. Stent deformation or fracture: Stent deformation was defined as multiple layers of struts seen within a single stent with loss of 3-dimentional stent integrity usually located at the proximal stent edge. Stent fracture included double layer of struts within a single stent with preserved 3-dimentional stent integrity usually located in the middle of the stent, assuming that stent struts were overlapped after fracture (overlap type), the dislocated or separated struts of a single stent (non-overlap type).
2. Untreated reference segment disease (categorical variable): A disease was defined as a lesion with >90° of arc of disease (>0.5mm of intimal thickness). A focal disease with untreated MLA <4.5 mm^2^ within 5 mm from the proximal and/or distal stent edges, otherwise it is categorized as a diffuse disease.
3. Lipidic plaque at the reference (categorical variable): Lipidic plaque is defined as a region with strong attenuation overlaid with a fibrous cap. Sub-classified by the amount of untreated lipid plaque, divided into 3 grades:

- Low (≤90° of lipid arc)
- Medium (>90°-<180° of lipid arc)
- High (≥180° of lipid arc)

*Note: If untreated reference segment disease with an MLA <4.5 mm^2^ was detected by operator assessment in either the proximal reference (inflow disease) or distal reference (outflow disease) segment lumen in the OCT-guided arm, the protocol directed that an additional stent must be placed to treat it, unless there were anatomic reasons that the disease should not be covered (e.g. diffuse distal disease or significant vessel tapering, etc.)*

1. Edge dissections (categorical variable): A flap of vessel wall within 5mm of stent edge

Edge dissections will be tabulated as:

- Major (%): ≥60 degrees of the circumference of the vessel at site of dissection and ≥3 mm in length
- Minor (%): any visible edge dissection <60 degrees of the circumference of the vessel or <3 mm in length

Edge dissections will be further classified as:

- Intimal (limited to the intima layer, i.e. not extending beyond the internal elastic lamina)
- Medial (extending into the media layer)
  - - Medial hematoma: a medial dissection but was distinguished by the appearance of blood accumulation within the medial space.
    - Medial non-hematoma: medial dissection without hematoma
- Adventitial (extending through the external elastic membrane/lamina)

Note: If a major edge dissection is detected by operator assessment in the OCT-guided arm, it is recommended that an additional stent be placed to cover the dissected segment, particularly if the site of dissection is at the distal stent edge.

**Definitions of Clinical Endpoints**

**Death (ARC-2 criteria)^1^:** All deaths are considered cardiac unless an unequivocal non-cardiac cause can be established. Specifically, any unexpected death even in patients with coexisting potentially fatal non-cardiac disease (e.g. cancer, infection) should be classified as cardiac.

**Cardiac death:**

Any death due to proximate cardiac cause (e.g. MI, low-output failure, fatal arrhythmia), unwitnessed death and death of unknown cause, all procedure related deaths including those related to concomitant treatment.

**Vascular death:**

Death due to non-coronary vascular causes such as cerebrovascular disease, pulmonary embolism, ruptured aortic aneurysm, dissecting aneurysm, or other vascular cause.

**Non-cardiovascular death:**

Any death not covered by the above definitions such as death caused by infection, malignancy, sepsis, pulmonary causes, accident, suicide or trauma.

**Ischemia-driven target lesion revascularization (ID-TLR)^1^:** A revascularization of target lesion is considered ischemia driven if associated with any of the following:

- Positive functional ischemia study including positive FFR, iFR, etc.
- Ischemic symptoms and angiographic diameter stenosis ≥50% by core laboratory QCA
- Angiographic diameter stenosis ≥ 70% by core laboratory QCA without angina or positive functional study

**Myocardial Infarction (Primary Protocol Definition)**

Periprocedural MI – Modified ARC-2 Definition^1^

Periprocedural myocardial infarction occurring within 48 hours after all percutaneous coronary intervention (PCI) and coronary artery bypass grafting (CABG) procedures:

Absolute rise (from baseline to within 48 hours of procedure) in cardiac troponin of ≥35x the 99th percentile URL (or ≥35x ULN if URL is not available) or in the absence of cardiac troponin, rise in CK-MB to ≥ 5x the 99th percentile URL (or ≥5x ULN if URL is not available). Note, cardiac troponin assessments are preferentially used if available; otherwise CK-MB may be used.

In addition, 1 (or more) of the following measures of myocardial ischemia must be present post-procedure:

- New significant Q waves (≥40 ms in duration and ≥1 mm deep in voltage in ≥2 contiguous leads) or equivalent
- Persistent flow-limiting angiographic complications in a major epicardial vessel or branch ≥1.5 mm in diameter present at the end of the PCI procedure (or during angiography performed to evaluate a post-CABG complication) as assessed at the angiographic core laboratory
- New substantial loss of viable myocardium on serial imaging

These assessments apply to patients: a) with baseline CK-MB or cardiac troponin levels ≤1x the 99th percentile URL (or ≤1x ULN if URL is not available); b) in whom the baseline biomarker is >1x the 99th percentile URL (or >1x ULN if URL is not available) and stable or falling; and c) with a single elevated baseline draw who have a chronic coronary syndrome (CCS). In the latter two groups (patients in whom the baseline is elevated and stable or falling and CCS patients with a single elevated baseline draw), the post-procedural troponin (or CK-MB) must rise above the most recent baseline by an increment of the values above (i.e. for troponin ≥35x the 99th percentile URL (or ≥35x ULN if URL is not available) or in the absence of cardiac troponin, for CK-MB ≥5x the 99th percentile URL (or ≥5x ULN if URL is not available).

These assessments do not apply to patients: a) in whom baseline CK-MB or troponin levels are elevated and rising; and b) with a single elevated baseline level who presented with a NSTEMI or STEMI in whom it is uncertain whether the peak has been reached. In such patients periprocedural MI will only be adjudicated if the troponin (or CK-MB) biomarker increases from the prior measure by the increments above, and at least 2 of the 3 above criteria for myocardial ischemia are present.

Spontaneous MI - 4^th^ Universal Definition of MI classification^2^

All MIs which are not peri-procedural are considered spontaneous MIs. Spontaneous myocardial infarctions are usually related to atherosclerotic plaque rupture, ulceration, fissuring, erosion, or dissection with resulting intraluminal thrombus in one or more of the coronary arteries leading to decreased myocardial blood flow or distal platelet emboli with ensuing myocyte necrosis. However, spontaneous MIs can also be due to an imbalance between supply and demand, or due to stent thrombosis, graft occlusion or other causes. Most patients with spontaneous MI have underlying severe CAD but on occasion non-obstructive or no CAD. Spontaneous MIs usually occur beyond 48 hours post-procedure but can occasionally occur within 48 hours of a revascularization procedure if the cause is clearly distinct from the index procedure (e.g. a stent thrombosis at 24 hours).

**Myocardial Infarction Relation to Target Vessel:**

Infarcts will be adjudicated according to anatomical origin based on review of coronary angiography performed at the time of the event. Those that cannot be clearly attributed to a particular vessel (target or non-target) either because the origin of the event on the angiogram is ambiguous or the angiogram was not performed will be considered as indeterminate vessel MI.

**Stent Thrombosis (definite or probable; modified ARC definitions):^1^**

Acute stent thrombosis (*): from completion of PCI procedure to 24 hours after stent implantation

Subacute stent thrombosis (*): >24 hours – 30 days after stent implantation

Late stent thrombosis (**): >30 days – 1 year after stent implantation

Very late stent thrombosis (**): >1 year after stent implantation

(*) acute or subacute can also be replaced by the term early stent thrombosis.

(**) including ‘primary’ as well as ‘secondary’ late stent thrombosis; ‘secondary’ late stent thrombosis is a stent thrombosis subsequent to a target lesion revascularization.

**Stent Thrombosis, Definite*:**

Definite stent thrombosis is considered to have occurred by either angiographic or pathological confirmation.

Angiographic confirmation of stent thrombosis

The presence of a thrombus that originates in the stent or in the segment 5 mm proximal or distal to the stent AND presence of at least 1 of the following criteria within a 48-hour time window:

- Acute onset of ischemic symptoms at rest
- New ischemic ECG changes that suggest acute ischemia
- Typical rise and fall in cardiac biomarkers (refer to definition of spontaneous MI)

*The incidental angiographic documentation of stent occlusion in the absence of clinical signs or symptoms is not considered a confirmed stent thrombosis (silent occlusion).

**Stent Thrombosis, Probable:**

Clinical definition of probable stent thrombosis is considered to have occurred after intracoronary stenting in the following cases:

- Any unexplained death within the first 30 days.
- Irrespective of the time after the index procedure, any MI that is related to documented acute ischemia in the territory of the implanted stent without angiographic confirmation of stent thrombosis and in the absence of any other obvious cause.

**Table S1. Angiographic characteristics and outcomes (core laboratory-assessed)**

|  | **All**  **(N=2128)** | **OCT guidance**  **(N=1056)** | **Angiography guidance**  **(N=1072)** | **P value** |
| --- | --- | --- | --- | --- |
| **Baseline** |  |  |  |  |
| Target vessel |  |  |  |  |
| Coronary artery |  |  |  |  |
| Left anterior descending | 1157/2127 (54.4) | 591/1055 (56.0) | 566/1072 (52.8) | 0.14 |
| Left circumflex | 397/2127 (18.7) | 192/1055 (18.2) | 205/1072 (19.1) | 0.58 |
| Right | 573/2127 (26.9) | 272/1055 (25.8) | 301/1072 (28.1) | 0.23 |
| TIMI III flow * | 1703/2115 (80.5) | 859/1053 (81.6) | 844/1062 (79.5) | 0.22 |
| Target lesion |  |  |  |  |
| Thrombus* | 156/2115 (7.4) | 75/1054 (7.1) | 81/1061 (7.6) | 0.65 |
| Calcification (severe)* | 649/2114 (30.7) | 328/1052 (31.2) | 321/1062 (30.2) | 0.63 |
| Reference vessel diameter, mm | 2.93 ± 0.42 | 2.94 ± 0.43 | 2.92 ± 0.41 | 0.17 |
| Minimal lumen diameter, mm | 0.88 ± 0.43 | 0.88 ± 0.43 | 0.89 ± 0.42 | 0.66 |
| Diameter stenosis, % | 69.7 ± 13.9 | 70.0 ± 14.0 | 69.5 ± 13.9 | 0.49 |
| Lesion length, mm | 31.9 ± 15.9 | 33.3 ± 15.7 | 30.6 ± 16.0 | <0.0001 |
| **Final post-PCI (target lesion)** |  |  |  |  |
| Reference vessel diameter, mm |  |  |  |  |
| In-stent | 2.96 ± 0.41 | 2.98 ± 0.44 | 2.94 ± 0.39 | 0.02 |
| In-segment | 2.87 ± 0.42 | 2.90± 0.44 | 2.85 ± 0.40 | 0.01 |
| Acute lumen gain, mm |  |  |  |  |
| In-stent | 1.75 ± 0.53 | 1.78 ± 0.54 | 1.73 ± 0.51 | 0.04 |
| In-segment | 1.56 ± 0.52 | 1.56 ± 0.53 | 1.55 ± 0.51 | 0.72 |
| Minimal lumen diameter, mm |  |  |  |  |
| In-stent | 2.64 ± 0.41 | 2.66 ± 0.43 | 2.62 ± 0.39 | 0.02 |
| In-segment | 2.44 ± 0.40 | 2.44 ± 0.41 | 2.44 ± 0.38 | 0.79 |
| Diameter stenosis, % |  |  |  |  |
| In-stent | 10.9 ± 6.4 | 10.9 ± 6.5 | 10.9 ± 6.2 | 0.77 |
| In-segment | 15.0 ± 6.8 | 15.6 ± 6.7 | 14.4 ± 6.8 | <0.0001 |
| TIMI III flow (per vessel)* | 2046/2101 (97.4) | 1020/1040 (98.1) | 1026/1061 (96.7) | 0.048 |

Data are presented as mean ± SD or n (%) or n/total n (%).

*Missing data is the result of incomplete visualization of the coronary arteries on angiography precluding the assessment of thrombus, calcification, and/or TIMI flow in selected patients.

OCT indicates optical coherence tomography; PCI indicates percutaneous coronary intervention; TIMI indicates Thrombolysis in Myocardial Infarction.

**Table S2. Medication use during study follow-up**

|  | **OCT guidance**  **(N=1056)** | **Angiography guidance (N=1072)** | **P value** |
| --- | --- | --- | --- |
| Discharge after index procedure |  |  |  |
| Any antiplatelet therapy | 1056 (100.0) | 1068/1071 (99.7) | 0.25 |
| Aspirin | 1028 (97.3) | 1045/1071 (97.6) | 0.74 |
| P2Y_12_ antagonist | 1049 (99.3) | 1063/1071 (99.3) | 0.82 |
| Dual antiplatelet therapy | 1021 (96.7) | 1040/1071 (97.1) | 0.58 |
| Beta-blocker | 734 (69.5) | 712/1071 (66.5) | 0.13 |
| Calcium-channel blocker | 239 (22.6) | 248/1071 (23.2) | 0.77 |
| ACE inhibitor or ARB | 686 (65.0) | 704/1071 (65.7) | 0.71 |
| Statin | 966 (91.5) | 984/1071 (91.9) | 0.74 |
| Long-term oral anticoagulant | 90 (8.5) | 67/1071 (6.3) | 0.046 |
| Vitamin K antagonist | 17 (1.6) | 9/1071 (0.8) | 0.11 |
| Direct-acting oral anticoagulant | 73 (6.9) | 58/1071 (5.4) | 0.15 |
| 30 days |  |  |  |
| Any antiplatelet therapy | 1031/1032 (99.9) | 1039/1042 (99.7) | 0.62 |
| Aspirin | 983/1032 (95.3) | 990/1042 (95.0) | 0.80 |
| P2Y_12_ antagonist | 1019/1032 (98.7) | 1028/1042 (98.7) | 0.87 |
| Dual antiplatelet therapy | 971/1032 (94.1) | 979/1042 (94.0) | 0.90 |
| Beta-blocker | 702/1032 (68.0) | 688/1042 (66.0) | 0.33 |
| Calcium-channel blocker | 227/1032 (22.0) | 220/1042 (21.1) | 0.62 |
| ACE inhibitor or ARB | 657/1032 (63.7) | 658/1042 (63.1) | 0.81 |
| Statin | 932/1032 (90.3) | 930/1042 (89.3) | 0.43 |
| Long-term oral anticoagulant | 89/1032 (8.6) | 74/1042 (7.1) | 0.20 |
| Vitamin K antagonist | 16/1032 (1.6) | 9/1042 (0.9) | 0.15 |
| Direct-acting oral anticoagulant | 73/1032 (7.1) | 65/1042 (6.2) | 0.45 |
| 1 year |  |  |  |
| Any antiplatelet therapy | 974/1006 (96.8) | 990/1013 (97.7) | 0.21 |
| Aspirin | 909/1006 (90.4) | 914/1013 (90.2) | 0.92 |
| P2Y_12_ antagonist | 674/1006 (67.0) | 703/1013 (69.4) | 0.25 |
| Dual antiplatelet therapy | 609/1006 (60.5) | 627/1013 (61.9) | 0.53 |
| Beta-blocker | 658/1006 (65.4) | 653/1013 (64.5) | 0.66 |
| Calcium-channel blocker | 206/1006 (20.5) | 212/1013 (20.9) | 0.80 |
| ACE inhibitor or ARB | 633/1006 (62.9) | 611/1013 (60.3) | 0.23 |
| Statin | 891/1006 (88.6) | 903/1013 (89.1) | 0.68 |
| Long-term oral anticoagulant | 100/1006 (9.9) | 74/1013 (7.3) | 0.03 |
| Vitamin K antagonist | 19/1006 (1.9) | 9/1013 (0.9) | 0.055 |
| Direct-acting oral anticoagulant | 81/1006 (8.1) | 65/1013 (6.4) | 0.16 |
| 2 years |  |  |  |
| Any antiplatelet therapy | 923/976 (94.6) | 922/972 (94.9) | 0.78 |
| Aspirin | 866/976 (88.7) | 864/972 (88.9) | 0.91 |
| P2Y_12_ antagonist | 333/976 (34.1) | 374/972 (38.5) | 0.046 |
| Dual antiplatelet therapy | 276/976 (28.3) | 316/972 (32.5) | 0.042 |
| Beta-blocker | 650/976 (66.6) | 614/972 (63.2) | 0.11 |
| Calcium-channel blocker | 210/976 (21.5) | 225/972 (23.1) | 0.39 |
| ACE inhibitor or ARB | 610/976 (62.5) | 598/972 (61.5) | 0.66 |
| Statin | 859/976 (88.0) | 855/972 (88.0) | 0.97 |
| Long-term oral anticoagulant | 106/976 (10.9) | 79/972 (8.1) | 0.04 |
| Vitamin K antagonist | 17/976 (1.7) | 9/972 (0.9) | 0.12 |
| Direct-acting oral anticoagulant | 89/976 (9.1) | 70/972 (7.2) | 0.12 |

Data are presented as n (%) or n/total n (%).

Analysis is based on available data and excludes missing subjects for whom the data field was not completed in the case report form. Denominators are provided where there is missing subject data.

ACE indicates angiotensin-converting enzyme; ARB indicates angiotensin-receptor blocker; OCT indicates optical coherence tomography.

**Table S3. OCT findings associated with cardiac death or target-vessel myocardial infarction (unadjusted)**

|  | **Number of events /number of patients at risk on day 0**  **(Kaplan-Meier estimates)** | **Hazard ratio (95% CI)** | **P value** |
| --- | --- | --- | --- |
| Minimal stent area, mm^2^ |  |  |  |
| < 5.24 (median) | 42/1063 (4.0) | Reference |  |
| ≥ 5.24 | 26/1065 (2.4) | 0.61 (0.38, 1.00) | 0.051 |
| Minimal stent expansion, % |  |  |  |
| < 79.1 (median) | 43/1063 (4.0) | Reference |  |
| ≥ 79.1 | 25/1065 (2.3) | 0.57 (0.35, 0.94) | 0.03 |
| Intra-stent flow area, mm^2^ |  |  |  |
| < 5.14 (median) | 44/1061 (4.1) | Reference |  |
| ≥ 5.14 | 24/1067 (2.2) | 0.54 (0.33, 0.88) | 0.01 |
| Total flow area, mm^2^ |  |  |  |
| < 5.23 (median) | 44/1058 (4.2) | Reference |  |
| ≥ 5.23 | 24/1070 (2.2) | 0.53 (0.33, 0.88) | 0.01 |
| Stent length, mm |  |  |  |
| < 33.4 (median) | 25/1056 (2.4) | Reference |  |
| ≥ 33.4 | 43/1072 (4.0) | 1.72 (1.05, 2.82) | 0.031 |
| Plaque or thrombus protrusion |  |  |  |
| None | 26/1034 (2.5) | Reference |  |
| Any | 42/1094 (3.8) | 1.53 (0.94, 2.49) | 0.09 |
| Major | 6/147 (4.1) | 1.61 (0.66, 3.90) | 0.29 |
| Minor | 36/947 (3.8) | 1.51 (0.91, 2.51) | 0.11 |
| Malapposition |  |  |  |
| None | 24/766 (3.1) | Reference |  |
| Any | 44/1362 (3.2) | 1.03 (0.63, 1.70) | 0.90 |
| Major | 21/549 (3.8) | 1.23 (0.68, 2.21) | 0.49 |
| Minor | 23/812 (2.8) | 0.90 (0.51, 1.60) | 0.73 |
| Stent deformation or fracture |  |  |  |
| None | 67/2107 (3.2) | Reference |  |
| Any | 1/21 (4.8) | 1.46 (0.20, 10.53) | 0.71 |
| Proximal or distal reference disease |  |  |  |
| None | 47/1608 (2.9) | Reference |  |
| Any | 21/513 (4.1) | 1.41 (0.84, 2.36) | 0.19 |
| Focal | 14/326 (4.3) | 1.48 (0.81, 2.68) | 0.20 |
| Diffuse | 8/210 (3.8) | 1.31 (0.62, 2.77) | 0.48 |
| Proximal reference disease |  |  |  |
| None | 46/1674 (2.7) | Reference |  |
| Any | 10/230 (4.3) | 1.60 (0.81, 3.18) | 0.18 |
| Focal | 8/170 (4.7) | 1.73 (0.82, 3.67) | 0.15 |
| Diffuse | 2/60 (3.3) | 1.24 (0.30, 5.10) | 0.77 |
| Distal reference disease |  |  |  |
| None | 53/1746 (3.0) | Reference |  |
| Any | 13/343 (3.8) | 1.25 (0.68, 2.30) | 0.46 |
| Focal | 7/177 (4.0) | 1.31 (0.60, 2.89) | 0.50 |
| Diffuse | 6/166 (3.6) | 1.19 (0.51, 2.78) | 0.68 |
| Proximal or distal reference lipidic plaque |  |  |  |
| None | 60/1767 (3.4) | Reference |  |
| Any | 8/355 (2.3) | 0.66 (0.32, 1.39) | 0.27 |
| Proximal reference lipidic plaque |  |  |  |
| None | 52/1683 (3.1) | Reference |  |
| Any | 4/238 (1.7) | 0.54 (0.20, 1.50) | 0.24 |
| Distal reference lipidic plaque |  |  |  |
| None | 62/1950 (3.2) | Reference |  |
| Any | 4/144 (2.8) | 0.87 (0.32, 2.40) | 0.79 |
| Proximal or distal reference dissection |  |  |  |
| None | 44/1430 (3.1) | Reference |  |
| Any | 24/695 (3.5) | 1.13 (0.69, 1.85) | 0.64 |
| Major | 3/66 (4.5) | 1.47 (0.45, 4.72) | 0.52 |
| Minor | 21/629 (3.3) | 1.09 (0.65, 1.83) | 0.75 |
| Intimal | 6/264 (2.3) | 0.74 (0.32, 1.74) | 0.49 |
| Medial | 19/460 (4.1) | 1.35 (0.79, 2.31) | 0.28 |
| Hematoma | 2/71 (2.8) | 0.91 (0.22, 3.75) | 0.89 |
| No hematoma | 17/398 (4.3) | 1.40 (0.80, 2.45) | 0.24 |
| Adventitial | 0/3 (0.0) | Not applicable | 0.99 |
| Proximal reference dissection, any |  |  |  |
| None | 42/1579 (2.7) | Reference |  |
| Any | 17/418 (4.1) | 1.54 (0.88, 2.70) | 0.13 |
| Major | 2/34 (5.9) | 2.23 (0.54, 9.20) | 0.27 |
| Minor | 15/384 (3.9) | 1.48 (0.82, 2.67) | 0.19 |
| Intimal | 6/188 (3.2) | 1.21 (0.51, 2.84) | 0.67 |
| Medial | 11/229 (4.8) | 1.82 (0.94, 3.53) | 0.08 |
| Hematoma | 0/25 (0.0) | Not applicable | 0.99 |
| No hematoma | 11/204 (5.4) | 2.05 (1.05, 3.98) | 0.03 |
| Adventitial | 0/1 (0.0) | Not applicable | 0.99 |
| Distal reference dissection |  |  |  |
| None | 56/1738 (3.2) | Reference |  |
| Any | 10/371 (2.7) | 0.84 (0.43, 1.65) | 0.61 |
| Major | 1/32 (3.1) | 0.95 (0.13, 6.85) | 0.96 |
| Minor | 9/339 (2.7) | 0.83 (0.41, 1.68) | 0.60 |
| Intimal | 0/89 (0.0) | Not applicable | 0.98 |
| Medial | 10/280 (3.6) | 1.12 (0.57, 2.19) | 0.75 |
| Hematoma | 2/46 (4.3) | 1.35 (0.33, 5.54) | 0.67 |
| No hematoma | 8/234 (3.4) | 1.07 (0.51, 2.24) | 0.86 |
| Adventitial | 0/2 (0.0) | Not applicable | 0.99 |

All subcategories are compared with the reference. Outcomes are presented as number of events during 2-year follow-up / number of the patients at risk on day 0 (Kaplan-Meier estimated event rates).

CI indicates confidence interval; OCT indicates optical coherence tomography.

**Table S4. OCT findings associated with ischemia-driven target lesion revascularization (unadjusted)**

|  | **Number of events /number of patients at risk on day 0**  **(Kaplan-Meier estimates)** | **Hazard ratio (95% CI)** | **P value** |
| --- | --- | --- | --- |
| Minimal stent area, mm^2^ |  |  |  |
| < 5.24 (median) | 58/1063 (5.5) | Reference |  |
| ≥ 5.24 | 29/1065 (2.7) | 0.49 (0.31, 0.77) | 0.002 |
| Minimal stent expansion, % |  |  |  |
| < 79.1 (median) | 53/1063 (5.0) | Reference |  |
| ≥ 79.1 | 34/1065 (3.2) | 0.63 (0.41, 0.97) | 0.04 |
| Intra-stent flow area, mm^2^ |  |  |  |
| < 5.14 (median) | 57/1061 (5.4) | Reference |  |
| ≥ 5.14 | 30/1067 (2.8) | 0.51 (0.33, 0.80) | 0.003 |
| Total flow area, mm^2^ |  |  |  |
| < 5.23 (median) | 57/1058 (5.4) | Reference |  |
| ≥ 5.23 | 30/1070 (2.8) | 0.51 (0.33, 0.80) | 0.003 |
| Stent length, mm |  |  |  |
| < 33.4 (median) | 43/1056 (4.1) | Reference |  |
| ≥ 33.4 | 44/1072 (4.1) | 1.02 (0.67, 1.55) | 0.93 |
| Plaque or thrombus protrusion |  |  |  |
| None | 43/1034 (4.2) | Reference |  |
| Any | 44/1094 (4.0) | 0.95 (0.63, 1.45) | 0.83 |
| Major | 10/147 (6.8) | 1.63 (0.82, 3.24) | 0.16 |
| Minor | 34/947 (3.6) | 0.85 (0.54, 1.33) | 0.48 |
| Malapposition |  |  |  |
| None | 36/766 (4.7) | Reference |  |
| Any | 51/1362 (3.7) | 0.79 (0.52, 1.22) | 0.29 |
| Major | 24/549 (4.4) | 0.93 (0.56, 1.57) | 0.80 |
| Minor | 27/812 (3.3) | 0.70 (0.43, 1.16) | 0.16 |
| Stent deformation or fracture |  |  |  |
| None | 85/2107 (4.0) | Reference |  |
| Any | 2/21 (9.5) | 2.27 (0.56, 9.21) | 0.25 |
| Proximal or distal reference disease |  |  |  |
| None | 58/1608 (3.6) | Reference |  |
| Any | 29/513 (5.7) | 1.59 (1.02, 2.48) | 0.04 |
| Focal | 18/326 (5.5) | 1.55 (0.91, 2.63) | 0.10 |
| Diffuse | 14/210 (6.7) | 1.87 (1.04, 3.36) | 0.04 |
| Proximal reference disease |  |  |  |
| None | 54/1674 (3.2) | Reference |  |
| Any | 15/230 (6.5) | 2.10 (1.19, 3.73) | 0.01 |
| Focal | 10/170 (5.9) | 1.89 (0.96, 3.71) | 0.06 |
| Diffuse | 5/60 (8.3) | 2.73 (1.09, 6.83) | 0.03 |
| Distal reference disease |  |  |  |
| None | 66/1746 (3.8) | Reference |  |
| Any | 19/343 (5.5) | 1.47 (0.88, 2.45) | 0.14 |
| Focal | 8/177 (4.5) | 1.19 (0.57, 2.49) | 0.64 |
| Diffuse | 11/166 (6.6) | 1.77 (0.93, 3.35) | 0.08 |
| Proximal or distal reference lipidic plaque |  |  |  |
| None | 76/1767 (4.3) | Reference |  |
| Any | 11/355 (3.1) | 0.72 (0.38, 1.35) | 0.30 |
| Proximal reference lipidic plaque |  |  |  |
| None | 64/1683 (3.8) | Reference |  |
| Any | 7/238 (2.9) | 0.77 (0.35, 1.67) | 0.51 |
| Distal reference lipidic plaque |  |  |  |
| None | 81/1950 (4.2) | Reference |  |
| Any | 4/144 (2.8) | 0.67 (0.24, 1.82) | 0.43 |
| Proximal or distal reference dissection |  |  |  |
| None | 54/1430 (3.8) | Reference |  |
| Any | 33/695 (4.7) | 1.27 (0.83, 1.96) | 0.28 |
| Major | 3/66 (4.5) | 1.20 (0.38, 3.85) | 0.75 |
| Minor | 30/629 (4.8) | 1.28 (0.82, 2.00) | 0.28 |
| Intimal | 13/264 (4.9) | 1.32 (0.72, 2.42) | 0.37 |
| Medial | 24/460 (5.2) | 1.40 (0.87, 2.27) | 0.17 |
| Hematoma | 6/71 (8.5) | 2.25 (0.97, 5.23) | 0.06 |
| No hematoma | 19/398 (4.8) | 1.28 (0.76, 2.17) | 0.35 |
| Adventitial | 0/3 (0.0) | Not applicable | 0.99 |
| Proximal reference dissection, any |  |  |  |
| None | 51/1579 (3.2) | Reference |  |
| Any | 25/418 (6.0) | 1.90 (1.18, 3.07) | 0.009 |
| Major | 2/34 (5.9) | 1.90 (0.46, 7.81) | 0.37 |
| Minor | 23/384 (6.0) | 1.90 (1.16, 3.11) | 0.01 |
| Intimal | 12/188 (6.4) | 2.02 (1.08, 3.80) | 0.03 |
| Medial | 13/229 (5.7) | 1.81 (0.98, 3.32) | 0.06 |
| Hematoma | 3/25 (12.0) | 3.82 (1.19, 12.23) | 0.02 |
| No hematoma | 10/204 (4.9) | 1.56 (0.79, 3.08) | 0.20 |
| Adventitial | 0/1 (0.0) | Not applicable | 0.99 |
| Distal reference dissection |  |  |  |
| None | 71/1738 (4.1) | Reference |  |
| Any | 14/371 (3.8) | 0.93 (0.52, 1.65) | 0.80 |
| Major | 1/32 (3.1) | 0.73 (0.10, 5.28) | 0.76 |
| Minor | 13/339 (3.8) | 0.95 (0.52, 1.71) | 0.86 |
| Intimal | 1/89 (1.1) | 0.27 (0.04, 1.97) | 0.20 |
| Medial | 13/280 (4.6) | 1.15 (0.63, 2.07) | 0.65 |
| Hematoma | 3/46 (6.5) | 1.59 (0.50, 5.05) | 0.43 |
| No hematoma | 10/234 (4.3) | 1.06 (0.55, 2.05) | 0.87 |
| Adventitial | 0/2 (0.0) | Not applicable | 0.98 |

All subcategories are compared with the reference. Outcomes are presented as number of events during 2-year follow-up / number of the patients at risk on day 0 (Kaplan-Meier estimated event rates).

CI indicates confidence interval; OCT indicates optical coherence tomography.

**Table S5. OCT findings associated with definite or probable stent thrombosis (unadjusted)**

|  | **Number of events /number of patients at risk on day 0**  **(Kaplan-Meier estimates)** | **Hazard ratio (95% CI)** | **P value** |
| --- | --- | --- | --- |
| Minimal stent area, mm^2^ |  |  |  |
| < 5.24 (median) | 11/1063 (1.0) | Reference |  |
| ≥ 5.24 | 7/1065 (0.7) | 0.63 (0.24, 1.63) | 0.34 |
| Minimal stent expansion, % |  |  |  |
| < 79.1 (median) | 11/1063 (1.0) | Reference |  |
| ≥ 79.1 | 7/1065 (0.7) | 0.63 (0.24, 1.63) | 0.34 |
| Intra-stent flow area, mm^2^ |  |  |  |
| < 5.14 (median) | 12/1061 (1.1) | Reference |  |
| ≥ 5.14 | 6/1067 (0.6) | 0.49 (0.19, 1.32) | 0.16 |
| Total flow area, mm^2^ |  |  |  |
| < 5.23 (median) | 12/1058 (1.1) | Reference |  |
| ≥ 5.23 | 6/1070 (0.6) | 0.49 (0.19, 1.31) | 0.16 |
| Stent length, mm |  |  |  |
| < 33.4 (median) | 5/1056 (0.5) | Reference |  |
| ≥ 33.4 | 13/1072 (1.2) | 2.58 (0.92, 7.24) | 0.07 |
| Plaque or thrombus protrusion |  |  |  |
| None | 7/1034 (0.7) | Reference |  |
| Any | 11/1094 (1.0) | 1.48 (0.57, 3.81) | 0.42 |
| Major | 2/147 (1.4) | 2.00 (0.42, 9.64) | 0.39 |
| Minor | 9/947 (1.0) | 1.40 (0.52, 3.75) | 0.51 |
| Malapposition |  |  |  |
| None | 6/766 (0.8) | Reference |  |
| Any | 12/1362 (0.9) | 1.12 (0.42, 2.99) | 0.82 |
| Major | 8/549 (1.5) | 1.87 (0.65, 5.38) | 0.25 |
| Minor | 4/812 (0.5) | 0.63 (0.18, 2.22) | 0.47 |
| Stent deformation or fracture |  |  |  |
| None | 18/2107 (0.9) | Reference |  |
| Any | 0/21 (0.0) | Not applicable | 0.99 |
| Proximal or distal reference disease |  |  |  |
| None | 11/1608 (0.7) | Reference |  |
| Any | 7/513 (1.4) | 2.01 (0.78, 5.18) | 0.15 |
| Focal | 5/326 (1.5) | 2.26 (0.78, 6.49) | 0.13 |
| Diffuse | 2/210 (1.0) | 1.40 (0.31, 6.32) | 0.66 |
| Proximal reference disease |  |  |  |
| None | 12/1674 (0.7) | Reference |  |
| Any | 3/230 (1.3) | 1.85 (0.52, 6.54) | 0.34 |
| Focal | 2/170 (1.2) | 1.66 (0.37, 7.41) | 0.51 |
| Diffuse | 1/60 (1.7) | 2.38(0.31, 18.30) | 0.40 |
| Distal reference disease |  |  |  |
| None | 12/1746 (0.7) | Reference |  |
| Any | 5/343 (1.5) | 2.13 (0.75, 6.04) | 0.16 |
| Focal | 4/177 (2.3) | 3.31 (1.07, 10.25) | 0.04 |
| Diffuse | 1/166 (0.6) | 0.88 (0.11, 6.75) | 0.90 |
| Proximal or distal reference lipidic plaque |  |  |  |
| None | 15/1767 (0.8) |  |  |
| Any | 3/355 (0.8) | 1.00 (0.29, 3.44) | 1.00 |
| Proximal reference lipidic plaque |  |  |  |
| None | 11/1683 (0.7) | Reference |  |
| Any | 3/238 (1.3) | 1.94 (0.54, 6.94) | 0.31 |
| Distal reference lipidic plaque |  |  |  |
| None | 17/1950 (0.9) | Reference |  |
| Any | 0/144 (0.0) | Not applicable | 0.99 |
| Proximal or distal reference dissection |  |  |  |
| None | 10/1430 (0.7) | Reference |  |
| Any | 8/695 (1.2) | 1.66 (0.65, 4.20) | 0.29 |
| Major | 0/66 (0.0) | Not applicable | 0.99 |
| Minor | 8/629 (1.3) | 1.83 (0.72, 4.64) | 0.20 |
| Intimal | 2/264 (0.8) | 1.09 (0.24, 4.98) | 0.91 |
| Medial | 6/460 (1.3) | 1.88 (0.68, 5.16) | 0.22 |
| Hematoma | 1/71 (1.4) | 2.02 (0.26, 15.76) | 0.50 |
| No hematoma | 5/398 (1.3) | 1.81 (0.62, 5.29) | 0.28 |
| Adventitial | 0/3 (0.0) | Not applicable | 0.99 |
| Proximal reference dissection, any |  |  |  |
| None | 10/1579 (0.6) | Reference |  |
| Any | 5/418 (1.2) | 1.90 (0.65, 5.57) | 0.24 |
| Major | 0/34 (0.0) | Not applicable | 0.99 |
| Minor | 5/384 (1.3) | 2.07 (0.71, 6.06) | 0.18 |
| Intimal | 2/188 (1.1) | 1.69 (0.37, 7.73) | 0.50 |
| Medial | 3/229 (1.3) | 2.08 (0.57, 7.57) | 0.26 |
| Hematoma | 0/25 (0.0) | Not applicable | 0.99 |
| No hematoma | 3/204 (1.5) | 2.35 (0.65, 8.52) | 0.20 |
| Adventitial | 0/1 (0.0) | Not applicable | 1.00 |
| Distal reference dissection |  |  |  |
| None | 14/1738 (0.8) | Reference |  |
| Any | 3/371 (0.8) | 1.01 (0.29, 3.51) | 0.99 |
| Major | 0/32 (0.0) | Not applicable | 0.99 |
| Minor | 3/339 (0.9) | 1.11 (0.32, 3.85) | 0.87 |
| Intimal | 0/89 (0.0) | Not applicable | 1.00 |
| Medial | 3/280 (1.1) | 1.34 (0.38, 4.65) | 0.65 |
| Hematoma | 1/46 (2.2) | 2.72 (0.36, 20.68) | 0.33 |
| No hematoma | 2/234 (0.9) | 1.06 (0.24, 4.69) | 0.93 |
| Adventitial | 0/2 (0.0) | Not applicable | 0.99 |

All subcategories are compared with the reference. Outcomes are presented as number of events during 2-year follow-up / number of the patients at risk on day 0 (Kaplan-Meier estimated event rates).

CI indicates confidence interval; OCT indicates optical coherence tomography.

**Table S6. Numbers and percentages of missing values in analyzed variables**

| **Variable** | **Number Missing** | **Percent Missing (%)** |
| --- | --- | --- |
| Minimal stent area, mm^2^ | 0 | 0.0 |
| Minimal stent expansion, % | 0 | 0.0 |
| Minimal intra-stent flow area, mm^2^ | 0 | 0.0 |
| Minimal total flow area, mm^2^ | 0 | 0.0 |
| Plaque or thrombus protrusion, any | 0 | 0.0 |
| Stent length, mm | 0 | 0.0 |
| Major | 0 | 0.0 |
| Minor | 0 | 0.0 |
| Malapposition, any | 0 | 0.0 |
| Major | 1 | 0.0 |
| Minor | 1 | 0.0 |
| Stent deformation or fracture | 0 | 0.0 |
| Proximal or distal reference disease, any | 7 | 0.3 |
| Focal | 75 | 3.5 |
| Diffuse | 79 | 3.7 |
| Proximal reference disease, any | 224 | 10.5 |
| Focal | 284 | 13.3 |
| Diffuse | 394 | 18.5 |
| Distal reference disease, any | 39 | 1.8 |
| Focal | 205 | 9.6 |
| Diffuse | 216 | 10.2 |
| Lipidic plaque | 6 | 0.3 |
| Proximal reference | 207 | 9.7 |
| Distal reference | 34 | 1.6 |
| Proximal or distal reference dissection, any | 3 | 0.1 |
| Major | 3 | 0.1 |
| Minor | 3 | 0.1 |
| Intimal | 3 | 0.1 |
| Medial | 3 | 0.1 |
| Hematoma | 3 | 0.1 |
| No hematoma | 3 | 0.1 |
| Adventitial | 3 | 0.1 |
| Proximal reference dissection, any | 131 | 6.2 |
| Major | 131 | 6.2 |
| Minor | 131 | 6.2 |
| Intimal | 131 | 6.2 |
| Medial | 131 | 6.2 |
| Hematoma | 131 | 6.2 |
| No hematoma | 131 | 6.2 |
| Adventitial | 131 | 6.2 |
| Distal reference dissection, any | 19 | 0.9 |
| Major | 19 | 0.9 |
| Minor | 19 | 0.9 |
| Intimal | 19 | 0.9 |
| Medial | 19 | 0.9 |
| Hematoma | 19 | 0.9 |
| No hematoma | 19 | 0.9 |
| Adventitial | 19 | 0.9 |

**Table S7. Association between intravascular imaging findings and clinical outcomes**

| **#** | **Study or first author, published year** | **Image** | **Study cohort** | **# of lesions / # of patients (Stent type)** | **Primary endpoints, other endpoint** | **# of primary composite events, follow-up duration** | **Imaging predictors**  **(Shown with underlines)** | **Comments** |
| --- | --- | --- | --- | --- | --- | --- | --- | --- |
| **Overall predictor analysis** | | | | | | | | |
| 1 | SIRIUS^1^, 2004 | IVUS | Multicenter, RCT, IVUS substudy | 122 lesions (59% SES, 41% BMS) / 122 patients | 8-month follow-up IVUS MLA>4mm^2^ | Not reported at 8-month | MSA, the cut-off value was 5.0mm^2^ for SES and 6.5mm^2^ for BMS | MSA/reference vessel area was less predictive for follow-up MLA. The study included only the lesions that had both index and follow-up IVUS. |
| 2 | Hong^2^, 2006 | IVUS | Single-center, registry | 543 lesions (100% SES) / 449 patients | Angiographic restenosis (DS≥50%), TLR | 21 angiographic restenosis per lesion (3.9%), 9 TLR at 6-month | MSA, the cut-off value was 5.5mm^2^  Stent length, the cut-off value was 40mm | No other IVUS findings other than MSA and reference MLA have been reported. |
| 3 | TAXUS^3^, 2009 | IVUS | Multicenter, RCTs, Pooled IVUS substudies | 1580 lesions (69% PES, 31% BMS) / 1580 patients | Angiographic restenosis (DS≥50%) | 259 angiographic restenosis (PES 10%, BMS 31%) at 9-month | MSA, the cut-off value was 5.7mm^2^ for PES and 6.4 mm^2^ for BMS | No other IVUS findings other than MSA have been reported. |
| 4 | HORIZONS-AMI^4^, 2012 | IVUS | Multicenter, RCT, IVUS substudy | 355 lesions / 318 STEMI patients (75% PES, 25% BMS) | Angiographic restenosis (DS≥50%) | 45 angiographic restenosis per lesion (12.7%) at 13-month | MSA | Stent malapposition, tissue protrusion, or stent edge dissection were not associated with the outcome. |
| 5 | Song^5^, 2014 | IVUS | Single-center, retrospective registry | 990 lesions (55% SES, 22% ZES-Resolute, 23% EES) / 912 patients | Angiographic restenosis (DS>50%) | 38 angiographic restenosis per lesion (3.8%) at 9-month | MSA, the cut-off value was 5.5mm^2^ for SES, 5.3mm^2^ for ZES, and 5.4mm^2^ for EES. | Vessel area and plaque burden at MSA sites were not associated with the outcome. |
| 6 | CLI-OPCI II^6^, 2015 | OCT | Multicenter, retrospective registry | 984 lesions (71% DES, 22% BMS, 7% BVS) / 832 patients | MACE (All-cause death, MI, or TLR) | 105 MACE events (24 deaths and 56 TLR) with a median of 0.9 year | In-stent or reference MLA<4.5mm^2^ Distal edge dissection (>200µm in thickness) | External validation for pre-specified criteria developed in prior CLI-OPCI registry to compare OCT vs angiography guided PCI^7^ |
| 7 | Soeda^8^, 2015 | OCT | Multicenter, registry | 900 lesions (85% DES, 15% BMS) / 786 patients | MACE (Cardiac death, TV-MI, TLR or ST) | 33 MACE events (31 TLR) at 1-year | MSA, the cut-off value was 5.0mm^2^ for DES and 5.6mm^2^ for BMS.  Irregular tissue protrusion (≥100µm of height with irregular surface) | Main enrolled countries were China, South Korea, or Japan. |
| 8 | ADAPT-DES^9^, 2016 | IVUS | Multicenter, prospective IVUS substudy | 2444 lesions (100% DES) / 2072 patients | MACE (Cardiac death, MI, or ST), TLR | 92 MACE events, 78 TLR at 2-year | Stent edge dissection and larger plaque burden at the in-stent MLA site were associated with more TLRs and tissue protrusion was associated with less TLR. | Overall, final in-stent MLA was large and in-stent MLA was not associated with TLR. |
| 9 | RESET and IVUS-XPL^10^, 2017 | IVUS | Multicenter, RCT, pooled IVUS cohorts | 804 lesions (100% EES) / 804 patients | MACE (Cardiac death, TL-MI, or TLR) | 24 MACE events at 1-year (22 TLR) | In-stent MLA, the cut-off value was 5.0mm^2^  The ratio of in-stent MLA to distal reference lumen area, the cut-off value was 1.0 | The lesions treated with long EES stent (≥28 mm) were included. |
| 10 | CLI-OPCI-LATE^11^, 2018 | OCT | Multicenter, retrospective registry | 1422 lesions (87% DES, 13% BMS) / 1211 patients | Cardiac death, TV-MI, or TLR | 144 MACE events (102 TLR) with a median of 2.3 years | In-stent or reference MLA<4.5mm^2^  Distal edge dissection (>200µm in thickness) | When the suboptimal morphology was combined, the association between suboptimal result and outcome was observed mainly within 1 year. |
| 11 | SYNTAX II^12^, 2019 | IVUS | Multicenter, prospective registry | 819 lesions (100% EES) / 367 patients with *de novo* 3 vessel disease | MACE (Cardiac death, TV-MI, or TLR) | 39 MACE events (27 TLR) at 2-year | MSA, the cut-off value was 5.2mm^2^ | Optimization was based on modified MUSIC criteria^13^. Reference MLA, stent symmetry, or malapposition were not associated with outcome |
| 12 | IVUS-XPL and ULTIMATE^14^, 2022 | IVUS | Multicenter, RCT, pooled IVUS cohorts | 1499 lesions (100% DES) / 1267 patients | MACE (Cardiac death, TL-MI, or ST) | 16 MACE events at 3-year | Meeting IVUS criteria was associated with lower MACE | Criteria met in 52% cases. IVUS-XPL (in-stent MLA>distal reference lumen area), ULTIMATE (MSA>5mm^2^ or >90% of distal reference MLA, reference plaque burden<50%, no medial edge dissection>3mm) |
| **Stent expansion using different definitions** | | | | | | | | |
| 13 | ILUMIEN I^15^, 2018 | OCT | Multicenter, prospective registry | 291 lesions (100% DES) /  289 patients | MACE (cardiac death, TV-MI, TLR, or ST) | 11 MACE events at 1-year | Minimum expansion index ≤73.3% (cut-off value) | Components of MACE have not been reported. Minimum stent expansion was defined as the smallest ratio of “actual lumen area / ideal lumen area” within the entire stent segment. |
| 14 | ADAPT DES^16^, 2021 | IVUS | Multicenter, prospective registry | 2140 lesions (100% DES) / 1831 patients with automatic IVUS pullbacks | Clinically driven TLR or definite ST | 72 TLRs or definite ST per lesion level at 2-year | MSA/vessel area at MSA site (%) ≤ 38.9% (cut-off value) | 10 stent expansion indices were tested including minimum expansion index (#13) and optimization criteria used in RCTs for imaging guidance, same cohort with #8 |
| 15 | IVUS-XPL and ULTIMATE^17^, 2021 | IVUS | Multicenter RCT, pooled IVUS cohorts | 1484 lesions (100% DES) / 1254 patients | MACE (Cardiac death, TL-MI, or ST) | 16 events (12 cardiac deaths) at 3- year | MSA<5.5 or 5.0mm^2^ (pre-specified criteria) | Small number of events and mainly cardiac death. Stent expansion ratio by average reference was available only in 70.5% of lesions, same cohort with #12 |
| 16 | CLI-OPCI^18^, 2022 | OCT | Multicenter, retrospective registry | 1422 lesions (87% DES, 13% BMS) / 1211 patients | MACE (Cardiac death, TV-MI, TLR, or ST) | 144 events (102 TLR) with a median of 2.3 years | In-stent MLA<4.5mm^2^, excessive stent expansion >110%, but not stent expansion<70%, <80%, or <90% | Stent expansion was defined as smaller ratio of in-stent MLA/corresponding, reference lumen area at proximal or distal halves of stent, same cohort with #10 |
| 17 | Lee^19^, 2023 | OCT | Single center, prospective registry | 1123 lesions (100% 2^nd^ generation DES) / 1071 patients | MACE (Cardiac death, TV-MI, TLR, or ST) | 44 MACE events (33 TLR) with a median of 3.5 years | MSA<5.0mm^2^ or excessive stent expansion by linear model ≥65% was associated with events |  |
| **Stent edge dissection** | | | | | | | | |
| 18 | ADAPT-DES^20^, 2016 | IVUS | Multicenter, prospective registry | 2433 lesions (100% DES) / 2062 patients | MACE (Cardiac death, MI, ST, or TLR) | 108 MACE events (58 TLR) at 2-year | Stent edge dissection (MLA within the dissection <5.1mm^2^ was associated with TLR) | Dissection arc or dissection length was not predictive, same cohort with #8 but including the lesions with stent edge IVUS |
| 19 | Van Zandvoort^21^, 2020 | OCT | Single center, retrospective registry | 295 lesions with untreated stent edge dissection / 261 patients | MACE (Cardiac death, TL-MI, or TLR) | 20 events (6.7%) at 1-year | Dissection length at either proximal or distal reference, the cut-off value was 3.6mm  At distal edge, cavity depth (luminal space between flap and surface of plaque)  At proximal edge, smaller reference lumen area | Evaluated dissection morphology includes longitudinal length, circumferential extension, thickness of dissection flap, dissection depth (intimal, medial, hematoma), cavity depth, and MLA within the dissection. Stent type was not listed, and enrollment duration was 2009-2017. |
| **Stent edge residual disease** | | | | | | | | |
| 20 | TAXUS^22^, 2009 | IVUS | Multicenter, RCTs, pooled IVUS substudies | 531 lesions / 531 patients (52% TAXUS and 45% BMS) | Angiographic edge restenosis (DS≥50%) | 27 lesions with angiographic edge restenosis (5.1%) at 9-month | Stent edge plaque burden, the cut-off value was 47.1% for TAXUS and 47.7% for BMS. | Same cohort with #3 but including the lesions with edge IVUS |
| 21 | Kang^23^, 2013 | IVUS | Single center, retrospective registry | 987 lesions (24% Endeavor, 25% Resolute, 51% EES) / 820 patients | Angiographic edge restenosis (DS≥50%) | 47/1668 stent edges had restenosis at 9 months in which 13 stent edge related TLR with a median of 2.5 years | Stent edge plaque burden, the cut-off value was 54.5%  Stent edge MLA, the cut-off value was 5.7mm^2^ | Among 3 DESs, the cut-off value for stent edge plaque burden was similar (51.9-56.4%), but the cut-off value for stent edge MLA varied (4.8-7.1mm^2^). |
| 22 | Ino^24^, 2016 | OCT | Single center, retrospective registry | 382 lesions (100% EES) / 319 patients | Angiographic edge restenosis (DS>50%), TLR | 32 lesions with angiographic edge restenosis in which 22 had TLR at 9-12 months | Lipidic plaque within 5mm from the stent edge, the cut-off value was 185°  MLA within 5mm from the stent edge, the cut-off value was 4.1mm^2^. | Other OCT morphological factors (Tissue protrusion, stent area at stent edge, etc.) were not associated with stent edge restenosis. |
| **Acute stent malapposition** | | | | | | | | |
| 23 | HORIZONS-AMI^25^, 2010 | IVUS | Multicenter, RCT, IVUS substudy | 263 lesions / 241 patients (76% PES, 24% BMS) | MACE (Death, MI, TLR, or ST) | 2 MI and 5 TLRs at 13-month | Malapposition was not associated with the outcome. | Only patients with both index and follow-up IVUS were included. Very low event rate, same cohort with #4 |
| 24 | ADAPT-DES^26^, 2016 | IVUS | Multicenter, prospective registry | 2446 lesions (100% DES) / 2072 patients | MACE (Cardiac death, MI, or ST), TLR | 92 MACE events, 78 TLRs at 2-year | Malapposition regardless of size was not associated with the outcome. | Detailed analysis for malapposition, same cohort with #8 |
| 25 | CLI-OPCI^27^, 2017 | OCT | Multicenter, retrospective registry, 2009-2013 | 1020 lesions / 864 patients | MACE (Death, TV-MI, TLR, or ST) | 106 MACE (27 definite ST and 3 probable ST) with a median of 0.8 year | Malapposition regardless of size (thickness or length) was not associated with the outcome. | Detailed analysis for malapposition, same cohort with #10, stent type was not listed. |
| 26 | Lee^28^, 2019 | OCT | Pooled 6 OCT RCTs to evaluated stent coverage | 444 lesions (100% DES) / 436 patients | MACE (Cardiac death, TL-MI, TLR, or ST) | 13 events (12 TLRs) at 5-year | Severe malapposition (defined as ≥400µm of distance or ≥ 1mm length) was not associated with the outcome | Severe malapposition was based on European Association of Percutaneous Cardiovascular Interventions (EAPCI) criteria^29^. |
| 27 | Kim^30^, 2022 | OCT | Single center, Registry | 1348 lesions (100% DES) / 1290 patients | MACE (cardiac death, TV-MI, TLR, or ST), major safety endpoint (cardiac death, TV-MI, or definite or probable ST) | 48 MACE events, and 15 major safety endpoints (11 STs) with a median of 3.6 years | Total malapposition volume (by Simpson’s rule using malapposition area and length) ≥7mm^3^ was associated with major safety endpoint.  MSA and total stent length were associated with MACE. | Early generation DES (Cypher and Taxus, or Endeavor) were used in 151 patients. Stent edge dissection or tissue protrusion was not associated with outcomes |
| **Late stent malapposition** | | | | | | | | |
| 28 | Hong^31^, 2004 | IVUS | Single center, | 683 lesions (76% SES, 24% PES) / 542 patients | MACE (Death, MI or TLR) after 6 months IVUS | Only one sudden death in non-LAM group with a mean of 11months after 6 months IVUS | Late acquired malapposition (LAM, not present at index procedure but newly appeared at 6-month follow-up IVUS) was not associated with subsequent outcome. | The lesions with both index and follow-up IVUS without event were included.  The prevalence of LAM was 12.1%. |
| 29 | Im E^32^, 2014 | OCT | Single center | 351 lesions (100% DES) / 351 patients | MACE (Cardiovascular death, TLR, TV-MI, or ST) | 28 MACE events with a median of 6.7 years from stent implantation | Late stent malapposition (persistent from acute malapposition or newly developed late acquired malapposition) was not associated with subsequent event | Patients with follow-up OCT without event within 1-year were included. |
| 30 | Lee^33^, 2020 | IVUS | Single center | 1261 patients (58% BMS and 42% 1^st^ generation DES) | MACE (cardiac death, TV-MI, TLR, ST), very late definite or probable ST | 225 MACE events (20 very late definite or probable ST) at 10-year | Late acquired stent malapposition of the first-generation DES, but no BMS was associated with very-late definite or probable ST and MACE | Late acquired stent malapposition (absent at index PCI but present at follow-up) was evaluated by symptom-unrelated 6-month IVUS. |
| **Predictor for early stent thrombosis** | | | | | | | | |
| 31 | Fujii^34^, 2005 | IVUS | Single center, retrospective, control matched | 15 patients with definite ST and 45 matched patients | Definite or probable ST | 14 definite ST and 1 probable ST | MSA, residual reference disease | Median time from index procedure to ST was 14 days. |
| 32 | Liu^35^, 2009 | IVUS | Single center, retrospective, matched control | 20 patients with definite ST, matched patients with ISR, 50 patients without event | Definite ST | 20 definite ST (17 early and 3 late ST) | Stent under-expansion by MSA, minimal stent expansion (MSA/reference lumen area) or mean stent expansion (mean stent area/reference lumen area) were more severe in ST than ISR. | SES was used in 74% and PES was used in 26%. |
| 33 | HORIZONS-AMI^36^, 2011 | IVUS | Multicenter, IVUS substudy, | 401 patients (76% PES, 24% BMS) | Definite or probable ST | 12 early ST and 4 late/very late ST at 3-year | Minimum flow area (stent area – tissue/thrombus protrusion)  Stent edge dissection | Only 12 early ST events without adjusted model |
| 34 | CLI-THRO study^37^, 2015 | OCT | Multicenter, retrospective registry, matched control | 62 patients | Definite subacute ST | 20 definite subacute ST and matched 42 patients without ST | MSA and Stent edge dissection were more prevalent in definite ST, but not malapposition. |  |

ADAPT-DES indicates Assessment of Dual Antiplatelet Therapy With Drug-Eluting Stents; BMS indicates bare metal stent; BVS indicates bioabsorbable vascular scaffold; CLI-OPCI indicates Centro per la Lotta contro l’Infarto-Optimisation of Percutaneous Coronary Intervention; DES indicates drug eluting stent; DS indicates diameter stenosis; EES indicates Everolimus-eluting stent; ISR indicates in-stent restenosis; IVUS indicates intravascular ultrasound; IVUS-XPL indicates The Impact of Intravascular Ultrasound Guidance on Outcomes of Xience Prime Stents in Long Lesions; MACE indicates major adverse cardiac event; HORIZONS-AMI indicates the Harmonizing Outcomes with Revascularization and Stents in Acute Myocardial Infarction; MI indicates myocardial infarction; MLA indicates minimal lumen area; MSA indicates minimal stent area; PES indicates Paclitaxel-eluting stent; RCT indicates randomized controlled trial; ULTIMATE indicates Intravascular Ultrasound Guided Drug Eluting Stents Implantation in All-Comers Coronary Lesions; SES indicates Sirolimus-eluting stent; TL indicates target lesion; TV indicates target vessel; OCT indicates optical coherence tomography; RESET indicates Real Safety and Efficacy of 3-month dual antiplatelet Therapy following Endeavor zotarolimus-eluting implantation; ST indicates stent thrombosis; SYNTAX indicates; ZES indicates zotarolimus-eluting stent.

**Table S8. Comparison of operator-detected vs. core laboratory-detected OCT findings**

| **OCT Finding** | **Operator Observation** | **Core Lab Observation** | **Agreement Rate (%)** |
| --- | --- | --- | --- |
| Expansion <90% (either proximal or distal) | 441 | 593 | 74.4% |
| Expansion <90% (proximal) | 386 | 531 | 72.7% |
| Expansion <90% (distal) | 133 | 179 | 74.3% |
| Untreated reference segment disease (focal or diffuse) | 159 | 226 | 70.4% |
| Any dissection | 135 | 317 | 42.6% |
| Any malapposition | 187 | 550 | 34.0% |
| Any plaque or tissue protrusion | 288 | 560 | 51.4% |

The agreement rate was calculated as operator/site observation divided by core lab observation.

**Figure S1. Distribution of OCT-derived minimal stent expansion and its relationship with target lesion failure (TLF) during 2-year follow-up.** Penalized spline analysis showing the 2-year TLF rate (green line) and 95% CI (green shading) in the pooled OCT and angiography guidance groups for each post-PCI MSE value as a continuous measure. The blue line and shading represents the penalized spline relationship of the hazard ratio and 95% confidence interval for TLF for each MSA value referenced to a HR of 1.0 for the median MSE of 79.1%.

**Figure S1. Distribution of OCT-derived minimal stent expansion and its relationship with target lesion failure (TLF) during 2-year follow-up.**


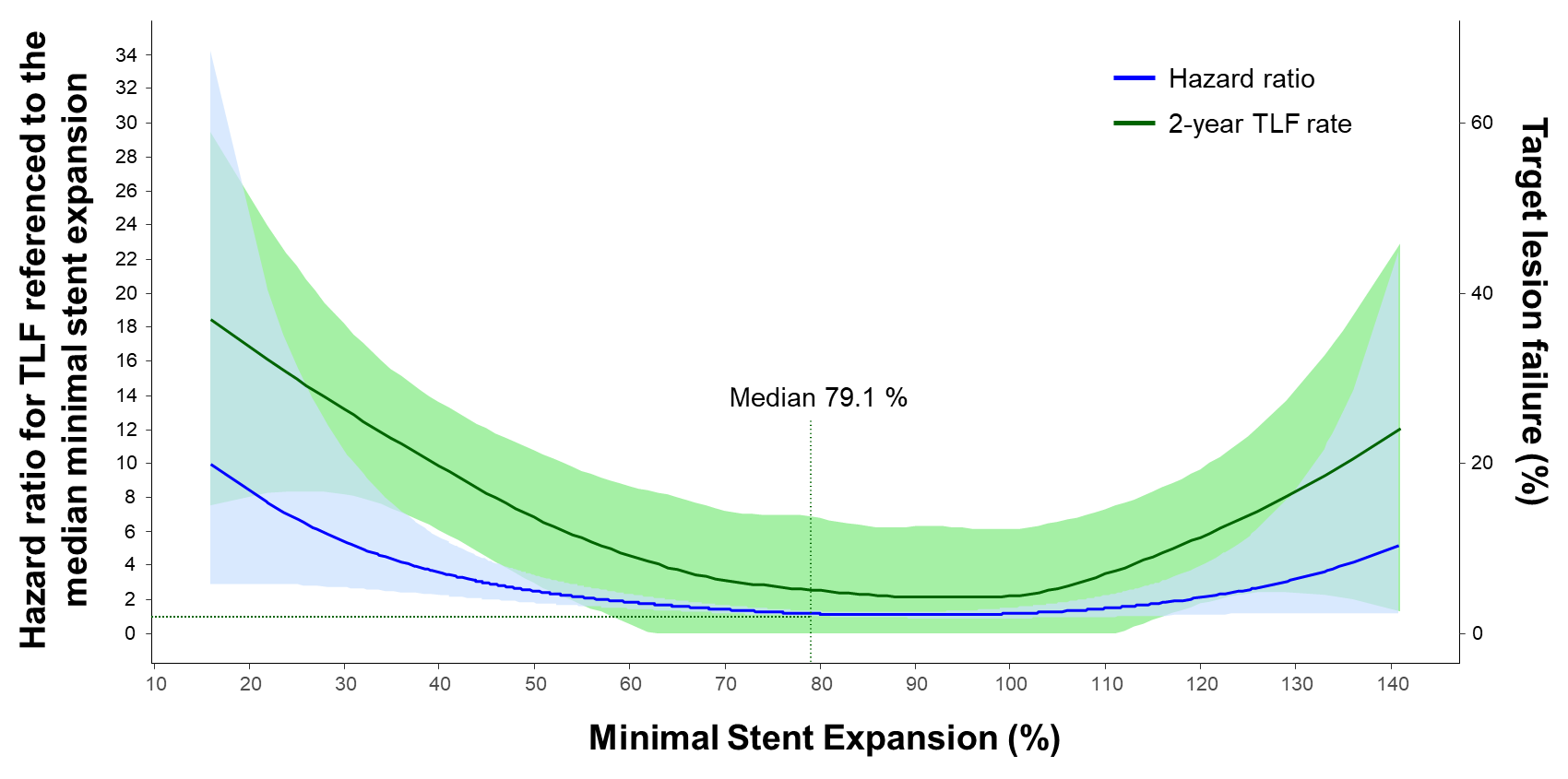


**References**

1. Sonoda S, Morino Y, Ako J, Terashima M, Hassan AH, Bonneau HN, Leon MB, Moses JW, Yock PG, Honda Y, Kuntz RE, Fitzgerald PJ; SIRIUS Investigators. [Impact of final stent dimensions on long-term results following sirolimus-eluting stent implantation: serial intravascular ultrasound analysis from the sirius trial.](https://pubmed-ncbi-nlm-nih-gov.ezproxy.cul.columbia.edu/15172398/) *J Am Coll Cardiol.* 2004;43:1959-1963.
2. Hong MK, Mintz GS, Lee CW, Park DW, Choi BR, Park KH, Kim YH, Cheong SS, Song JK, Kim JJ, Park SW, Park SJ. [Intravascular ultrasound predictors of angiographic restenosis after sirolimus-eluting stent implantation.](https://pubmed-ncbi-nlm-nih-gov.ezproxy.cul.columbia.edu/16682378/) *Eur Heart J.* 2006;27:1305-1310.
3. Doi H, Maehara A, Mintz GS, Yu A, Wang H, Mandinov L, Popma JJ, Ellis SG, Grube E, Dawkins KD, Weissman NJ, Turco MA, Ormiston JA, Stone GW. [Impact of post-intervention minimal stent area on 9-month follow-up patency of paclitaxel-eluting stents: an integrated intravascular ultrasound analysis from the TAXUS IV, V, and VI and TAXUS ATLAS Workhorse, Long Lesion, and Direct Stent Trials.](https://pubmed-ncbi-nlm-nih-gov.ezproxy.cul.columbia.edu/20129555/) *JACC Cardiovasc Interv.* 2009;2:1269-1275.
4. Choi SY, Maehara A, Cristea E, Witzenbichler B, Guagliumi G, Brodie B, Kellett MA Jr, Dressler O, Lansky AJ, Parise H, Mehran R, Mintz GS, Stone GW. [Usefulness of minimum stent cross sectional area as a predictor of angiographic restenosis after primary percutaneous coronary intervention in acute myocardial infarction (from the HORIZONS-AMI Trial IVUS substudy).](https://pubmed-ncbi-nlm-nih-gov.ezproxy.cul.columbia.edu/22118823/) *Am J Cardiol.* 2012;109:455-460.
5. Song HG, Kang SJ, Ahn JM, Kim WJ, Lee JY, Park DW, Lee SW, Kim YH, Lee CW, Park SW, Park SJ. [Intravascular ultrasound assessment of optimal stent area to prevent in-stent restenosis after zotarolimus-, everolimus-, and sirolimus-eluting stent implantation.](https://pubmed-ncbi-nlm-nih-gov.ezproxy.cul.columbia.edu/22815193/) *Catheter Cardiovasc Interv.* 2014;83:873-878.
6. Prati F, Romagnoli E, Burzotta F, Limbruno U, Gatto L, La Manna A, Versaci F, Marco V, Di Vito L, Imola F, Paoletti G, Trani C, Tamburino C, Tavazzi L, Mintz GS. [Clinical Impact of OCT Findings During PCI: The CLI-OPCI II Study.](https://pubmed-ncbi-nlm-nih-gov.ezproxy.cul.columbia.edu/26563859/) *JACC Cardiovasc Imaging.* 2015;8:1297-1305.
7. Prati F, Di Vito L, Biondi-Zoccai G, Occhipinti M, La Manna A, Tamburino C, Burzotta F, Trani C, Porto I, Ramazzotti V, Imola F, Manzoli A, Materia L, Cremonesi A, Albertucci M.. Angiography alone versus angiography plus optical coherence tomography to guide decision-making during percutaneous coronary intervention: the Centro per la Lotta contro l’Infarto-Optimisation of Percutaneous Coronary Intervention (CLI-OPCI) study. *EuroIntervention* 2012;8:823–829.
8. Soeda T, Uemura S, Park SJ, Jang Y, Lee S, Cho JM, Kim SJ, Vergallo R, Minami Y, Ong DS, Gao L, Lee H, Zhang S, Yu B, Saito Y, Jang IK. [Incidence and Clinical Significance of Poststent Optical Coherence Tomography Findings: One-Year Follow-Up Study From a Multicenter Registry.](https://pubmed-ncbi-nlm-nih-gov.ezproxy.cul.columbia.edu/26162917/) *Circulation.* 2015;132:1020-1029.
9. Qiu F, Mintz GS, Witzenbichler B, Metzger DC, Rinaldi MJ, Duffy PL, Weisz G, Stuckey TD, Brodie BR, Parvataneni R, Kirtane AJ, Stone GW, Maehara A. [Prevalence and Clinical Impact of Tissue Protrusion After Stent Implantation: An ADAPT-DES Intravascular Ultrasound Substudy.](https://pubmed-ncbi-nlm-nih-gov.ezproxy.cul.columbia.edu/27478119/) *JACC Cardiovasc Interv*. 2016;9:1499-1507.
10. Lee SY, Shin DH, Kim JS, Kim BK, Ko YG, Choi D, Jang Y, Hong MK. [Intravascular Ultrasound Predictors of Major Adverse Cardiovascular Events After Implantation of Everolimus-eluting Stents for Long Coronary Lesions.](https://pubmed-ncbi-nlm-nih-gov.ezproxy.cul.columbia.edu/27789170/) *Rev Esp Cardiol.* 2017;70:88-95.
11. Prati F, Romagnoli E, La Manna A, Burzotta F, Gatto L, Marco V, Fineschi M, Fabbiocchi F, Versaci F, Trani C, Tamburino C, Alfonso F, Mintz GS. [Long-term consequences of optical coherence tomography findings during percutaneous coronary intervention: the Centro Per La Lotta Contro L'infarto - Optimization Of Percutaneous Coronary Intervention (CLI-OPCI) LATE study.](https://pubmed-ncbi-nlm-nih-gov.ezproxy.cul.columbia.edu/29633940/) *EuroIntervention.* 2018;14:e443-e451.
12. Katagiri Y, De Maria GL, Kogame N, Chichareon P, Takahashi K, Chang CC, Modolo R, Walsh S, Sabate M, Davies J, Lesiak M, Moreno R, Cruz-Gonzalez I, West NEJ, Piek JJ, Wykrzykowska JJ, Farooq V, Escaned J, Banning AP, Onuma Y, Serruys PW. [Impact of post-procedural minimal stent area on 2-year clinical outcomes in the SYNTAX II trial.](https://pubmed-ncbi-nlm-nih-gov.ezproxy.cul.columbia.edu/30702187/) *Catheter Cardiovasc Interv.* 2019;93:E225-E234.
13. Escaned J, Collet C, Ryan N, De Maria GL, Walsh S, Sabate M, Davies J, Lesiak M, Moreno R, Cruz-Gonzalez I, Hoole SP, Ej West N, Piek JJ, Zaman A, Fath-Ordoubadi F, Stables RH, Appleby C, van Mieghem N, van Geuns RJ, Uren N, Zueco J, Buszman P, Iñiguez A, Goicolea J, Hildick-Smith D, Ochala A, Dudek D, Hanratty C, Cavalcante R, Kappetein AP, Taggart DP, van Es GA, Morel MA, de Vries T, Onuma Y, Farooq V, Serruys PW, Banning AP. [Clinical outcomes of state-of-the-art percutaneous coronary revascularization in patients with de novo three vessel disease: 1-year results of the SYNTAX II study.](https://pubmed-ncbi-nlm-nih-gov.ezproxy.cul.columbia.edu/29020367/) *Eur Heart J.* 2017;38:3124-3134.
14. Hong SJ, Zhang JJ, Mintz GS, Ahn CM, Kim JS, Kim BK, Ko YG, Choi D, Jang Y, Kan J, Pan T, Gao X, Ge Z, Chen SL, Hong MK. [Improved 3-Year Cardiac Survival After IVUS-Guided Long DES Implantation: A Patient-Level Analysis From 2 Randomized Trials.](https://pubmed-ncbi-nlm-nih-gov.ezproxy.cul.columbia.edu/35057991/) *JACC Cardiovasc Interv.* 2022;15:208-216.
15. Nakamura D, Wijns W, Price MJ, Jones MR, Barbato E, Akasaka T, Lee SW, Patel SM, Nishino S, Wang W, Gopinath A, Attizzani GF, Holmes D, Bezerra HG. [New Volumetric Analysis Method for Stent Expansion and its Correlation With Final Fractional Flow Reserve and Clinical Outcome: An ILUMIEN I Substudy.](https://pubmed-ncbi-nlm-nih-gov.ezproxy.cul.columbia.edu/30093052/) *JACC Cardiovasc Interv.* 2018;11:1467-1478.
16. Fujimura T, Matsumura M, Witzenbichler B, Metzger DC, Rinaldi MJ, Duffy PL, Weisz G, Stuckey TD, Ali ZA, Zhou Z, Mintz GS, Stone GW, Maehara A. [Stent Expansion Indexes to Predict Clinical Outcomes: An IVUS Substudy From ADAPT-DES.](https://pubmed-ncbi-nlm-nih-gov.ezproxy.cul.columbia.edu/34353595/) *JACC Cardiovasc Interv.* 2021;14:1639-1650.
17. Lee YJ, Zhang JJ, Mintz GS, Hong SJ, Ahn CM, Kim JS, Kim BK, Ko YG, Choi D, Jang Y, Kan J, Pan T, Gao X, Ge Z, Chen SL, Hong MK. Impact of Intravascular Ultrasound-Guided Optimal Stent Expansion on 3-Year Hard Clinical Outcomes. *Circ Cardiovasc Interv.* 2021;14:e011124. doi: [10.1161/CIRCINTERVENTIONS.121.011124](https://doi-org.ezproxy.cul.columbia.edu/10.1161/circinterventions.121.011124)
18. Romagnoli E, Ramazzotti V, Burzotta F, Gatto L, Marco V, Paoletti G, Biondi-Zoccai G, Alfonso F, Crea F, Trani C, Prati F. [Definition of Optimal Optical Coherence Tomography-Based Stent Expansion Criteria: In-Stent Minimum Lumen Area Versus Residual Stent Underexpansion.](https://pubmed-ncbi-nlm-nih-gov.ezproxy.cul.columbia.edu/36126136/) *Circ Cardiovasc Interv.* 2022 doi: 10.1161/CIRCINTERVENTIONS.121.011496.
19. Lee B, Baraki TG, Kim BG, Lee YJ, Lee SJ, Hong SJ, Ahn CM, Shin DH, Kim BK, Ko YG, Choi D, Hong MK, Jang Y, Kim JS. [Stent expansion evaluated by optical coherence tomography and subsequent outcomes.](https://pubmed-ncbi-nlm-nih-gov.ezproxy.cul.columbia.edu/36882449/) *Sci Rep.* 2023;13:3781. doi: 10.1038/s41598-023-30717-6.
20. Kobayashi N, Mintz GS, Witzenbichler B, Metzger DC, Rinaldi MJ, Duffy PL, Weisz G, Stuckey TD, Brodie BR, Parvataneni R, Kirtane AJ, Stone GW, Maehara A. [Prevalence, Features, and Prognostic Importance of Edge Dissection After Drug-Eluting Stent Implantation: An ADAPT-DES Intravascular Ultrasound Substudy.](https://pubmed-ncbi-nlm-nih-gov.ezproxy.cul.columbia.edu/27402854/) *Circ Cardiovasc Interv.* 2016;9:e003553. doi: 10.1161/CIRCINTERVENTIONS.115.003553.
21. van Zandvoort LJC, Tomaniak M, Tovar Forero MN, Masdjedi K, Visseren L, Witberg K, Ligthart J, Kardys I, Lemmert ME, Diletti R, Wilschut J, de Jaegere P, Zijlstra F, Van Mieghem NM, Daemen J. [Predictors for Clinical Outcome of Untreated Stent Edge Dissections as Detected by Optical Coherence Tomography.](https://pubmed-ncbi-nlm-nih-gov.ezproxy.cul.columbia.edu/32089001/) *Circ Cardiovasc Interv.* 2020;13:e008685. doi: 10.1161/CIRCINTERVENTIONS.119.008685.
22. Liu J, Maehara A, Mintz GS, Weissman NJ, Yu A, Wang H, Mandinov L, Popma JJ, Ellis SG, Grube E, Dawkins KD, Stone GW. [An integrated TAXUS IV, V, and VI intravascular ultrasound analysis of the predictors of edge restenosis after bare metal or paclitaxel-eluting stents.](https://pubmed-ncbi-nlm-nih-gov.ezproxy.cul.columbia.edu/19195510/) *Am J Cardiol.* 2009;103:501-506.
23. Kang SJ, Cho YR, Park GM, Ahn JM, Kim WJ, Lee JY, Park DW, Lee SW, Kim YH, Lee CW, Mintz GS, Park SW, Park SJ. [Intravascular ultrasound predictors for edge restenosis after newer generation drug-eluting stent implantation.](https://pubmed-ncbi-nlm-nih-gov.ezproxy.cul.columbia.edu/23433757/) *Am J Cardiol.* 2013;111:1408-1414.
24. Ino Y, Kubo T, Matsuo Y, Yamaguchi T, Shiono Y, Shimamura K, Katayama Y, Nakamura T, Aoki H, Taruya A, Nishiguchi T, Satogami K, Yamano T, Kameyama T, Orii M, Ota S, Kuroi A, Kitabata H, Tanaka A, Hozumi T, Akasaka T. [Optical Coherence Tomography Predictors for Edge Restenosis After Everolimus-Eluting Stent Implantation.](https://pubmed-ncbi-nlm-nih-gov.ezproxy.cul.columbia.edu/27688261/) *Circ Cardiovasc Interv.* 2016;9:e004231. doi: 10.1161/CIRCINTERVENTIONS.116.004231.
25. Guo N, Maehara A, Mintz GS, He Y, Xu K, Wu X, Lansky AJ, Witzenbichler B, Guagliumi G, Brodie B, Kellett MA Jr, Dressler O, Parise H, Mehran R, Stone GW. [Incidence, mechanisms, predictors, and clinical impact of acute and late stent malapposition after primary intervention in patients with acute myocardial infarction: an intravascular ultrasound substudy of the Harmonizing Outcomes with Revascularization and Stents in Acute Myocardial Infarction (HORIZONS-AMI) trial.](https://pubmed-ncbi-nlm-nih-gov.ezproxy.cul.columbia.edu/20805433/) *Circulation.* 2010;122:1077-1084.
26. Wang B, Mintz GS, Witzenbichler B, Souza CF, Metzger DC, Rinaldi MJ, Duffy PL, Weisz G, Stuckey TD, Brodie BR, Matsumura M, Yamamoto MH, Parvataneni R, Kirtane AJ, Stone GW, Maehara A. [Predictors and Long-Term Clinical Impact of Acute Stent Malapposition: An Assessment of Dual Antiplatelet Therapy With Drug-Eluting Stents (ADAPT-DES) Intravascular Ultrasound Substudy.](https://pubmed-ncbi-nlm-nih-gov.ezproxy.cul.columbia.edu/28007741/) *J Am Heart Assoc.* 2016;5:e004438. doi: 10.1161/JAHA.116.004438.
27. Romagnoli E, Gatto L, La Manna A, Burzotta F, Taglieri N, Saia F, Amico F, Marco V, Ramazzotti V, Di Giorgio A, Di Vito L, Boi A, Contarini M, Castriota F, Mintz GS, Prati F. [Role of residual acute stent malapposition in percutaneous coronary interventions.](https://pubmed-ncbi-nlm-nih-gov.ezproxy.cul.columbia.edu/28295990/) *Catheter Cardiovasc Interv.* 2017;90:566-575.
28. Lee SY, Im E, Hong SJ, Ahn CM, Kim JS, Kim BK, Ko YG, Choi D, Jang Y, Hong MK.J Am Heart Assoc. [Severe Acute Stent Malapposition After Drug-Eluting Stent Implantation: Effects on Long-Term Clinical Outcomes.](https://pubmed-ncbi-nlm-nih-gov.ezproxy.cul.columbia.edu/31237187/) *J Am Heart Assoc.* 2019;8:e012800. doi: 10.1161/JAHA.119.012800
29. Räber L, Mintz GS, Koskinas KC, Johnson TW, Holm NR, Onuma Y, Radu MD, Joner M, Yu B, Jia H, Meneveau N, de la Torre Hernandez JM, Escaned J, Hill J, Prati F, Colombo A, Di Mario C, Regar E, Capodanno D, Wijns W, Byrne RA, Guagliumi G. [Clinical use of intracoronary imaging. Part 1: guidance and optimization of coronary interventions. An expert consensus document of the European Association of Percutaneous Cardiovascular Interventions.](https://pubmed-ncbi-nlm-nih-gov.ezproxy.cul.columbia.edu/29939149/) *EuroIntervention.* 2018;14:656-677.
30. Kim BG, Kachel M, Kim JS, Guagliumi G, Kim C, Kim IS, Lee YJ, Lee OH, Byun YS, Kim BO, Milewski K, Lee SJ, Hong SJ, Ahn CM, Shin DH, Kim BK, Ko YG, Choi D, Jang Y. [Clinical Implications of Poststent Optical Coherence Tomographic Findings: Severe Malapposition and Cardiac Events.](https://pubmed-ncbi-nlm-nih-gov.ezproxy.cul.columbia.edu/34023255/) *JACC Cardiovasc Imaging*. 2022;15:126-137.
31. Hong MK, Mintz GS, Lee CW, Kim YH, Lee SW, Song JM, Han KH, Kang DH, Song JK, Kim JJ, Park SW, Park SJ. Incidence, mechanism, predictors, and long-term prognosis of late stent malapposition after bare-metal stent implantation. *Circulation.* 2004;109:881-886.
32. Im E, Kim BK, Ko YG, Shin DH, Kim JS, Choi D, Jang Y, Hong MK. [Incidences, predictors, and clinical outcomes of acute and late stent malapposition detected by optical coherence tomography after drug-eluting stent implantation.](https://pubmed-ncbi-nlm-nih-gov.ezproxy.cul.columbia.edu/24425586/) *Circ Cardiovasc Interv.* 2014;7:88-96
33. Lee SY, Ahn JM, Mintz GS, Hong SJ, Ahn CM, Park DW, Kim JS, Kim BK, Ko YG, Choi D, Jang Y, Park SJ, Hong MK. [Ten-Year Clinical Outcomes of Late-Acquired Stent Malapposition After Coronary Stent Implantation.](https://pubmed-ncbi-nlm-nih-gov.ezproxy.cul.columbia.edu/31766872/) *Arterioscler Thromb Vasc Biol.* 2020;40:288-295.
34. Fujii K, Carlier SG, Mintz GS, Yang YM, Moussa I, Weisz G, Dangas G, Mehran R, Lansky AJ, Kreps EM, Collins M, Stone GW, Moses JW, Leon MB. [Stent underexpansion and residual reference segment stenosis are related to stent thrombosis after sirolimus-eluting stent implantation: an intravascular ultrasound study.](https://pubmed-ncbi-nlm-nih-gov.ezproxy.cul.columbia.edu/15808753/) *J Am Coll Cardiol.* 2005;45:995-998.
35. Liu X, Doi H, Maehara A, Mintz GS, Costa Jde R Jr, Sano K, Weisz G, Dangas GD, Lansky AJ, Kreps EM, Collins M, Fahy M, Stone GW, Moses JW, Leon MB, Mehran R. [A volumetric intravascular ultrasound comparison of early drug-eluting stent thrombosis versus restenosis.](https://pubmed-ncbi-nlm-nih-gov.ezproxy.cul.columbia.edu/19463466/) *JACC Cardiovasc Interv.* 2009;2:428-434.
36. Choi SY, Witzenbichler B, Maehara A, Lansky AJ, Guagliumi G, Brodie B, Kellett MA Jr, Dressler O, Parise H, Mehran R, Dangas GD, Mintz GS, Stone GW. [Intravascular ultrasound findings of early stent thrombosis after primary percutaneous intervention in acute myocardial infarction: a Harmonizing Outcomes with Revascularization and Stents in Acute Myocardial Infarction (HORIZONS-AMI) substudy.](https://pubmed-ncbi-nlm-nih-gov.ezproxy.cul.columbia.edu/21586693/) *Circ Cardiovasc Interv.* 2011;4:239-247.
37. Prati F, Kodama T, Romagnoli E, Gatto L, Di Vito L, Ramazzotti V, Chisari A, Marco V, Cremonesi A, Parodi G, Albertucci M, Alfonso F. [Suboptimal stent deployment is associated with subacute stent thrombosis: optical coherence tomography insights from a multicenter matched study. From the CLI Foundation investigators: the CLI-THRO study.](https://pubmed-ncbi-nlm-nih-gov.ezproxy.cul.columbia.edu/25641534/) *Am Heart J.* 2015;169:249-256.
